# Traces of Semantization, from Episodic to Semantic Memory in a Spiking Cortical Network Model

**DOI:** 10.1523/ENEURO.0062-22.2022

**Published:** 2022-07-29

**Authors:** Nikolaos Chrysanthidis, Florian Fiebig, Anders Lansner, Pawel Herman

**Affiliations:** 1 Division of Computational Science and Technology, School of Electrical Engineering and Computer Science, KTH Royal Institute of Technology, Stockholm 10044, Sweden; 2 Department of Mathematics, Stockholm University, Stockholm 10691, Sweden; 3 Digital Futures, Stockholm 10044, Sweden; 4 Swedish e-Science Research Centre, Stockholm 10044, Sweden

**Keywords:** Bayesian–Hebbian plasticity, BCPNN, episodic memory, semantization, spiking cortical memory model, STDP

## Abstract

Episodic memory is a recollection of past personal experiences associated with particular times and places. This kind of memory is commonly subject to loss of contextual information or “semantization,” which gradually decouples the encoded memory items from their associated contexts while transforming them into semantic or gist-like representations. Novel extensions to the classical Remember/Know (R/K) behavioral paradigm attribute the loss of episodicity to multiple exposures of an item in different contexts. Despite recent advancements explaining semantization at a behavioral level, the underlying neural mechanisms remain poorly understood. In this study, we suggest and evaluate a novel hypothesis proposing that Bayesian–Hebbian synaptic plasticity mechanisms might cause semantization of episodic memory. We implement a cortical spiking neural network model with a Bayesian–Hebbian learning rule called Bayesian Confidence Propagation Neural Network (BCPNN), which captures the semantization phenomenon and offers a mechanistic explanation for it. Encoding items across multiple contexts leads to item-context decoupling akin to semantization. We compare BCPNN plasticity with the more commonly used spike-timing-dependent plasticity (STDP) learning rule in the same episodic memory task. Unlike BCPNN, STDP does not explain the decontextualization process. We further examine how selective plasticity modulation of isolated salient events may enhance preferential retention and resistance to semantization. Our model reproduces important features of episodicity on behavioral timescales under various biological constraints while also offering a novel neural and synaptic explanation for semantization, thereby casting new light on the interplay between episodic and semantic memory processes.

## Significance Statement

Remembering single episodes is a fundamental attribute of cognition. Difficulties recollecting contextual information is a key sign of episodic memory loss or semantization. Behavioral studies demonstrate that semantization of episodic memory can occur rapidly, yet the neural mechanisms underlying this effect are insufficiently investigated. In line with recent behavioral findings, we show that multiple stimulus exposures in different contexts may advance item-context decoupling. We suggest a Bayesian–Hebbian synaptic plasticity hypothesis of memory semantization and further show that a transient modulation of plasticity during salient events may disrupt the decontextualization process by strengthening memory traces, and thus, enhancing preferential retention. The proposed cortical network-of-networks model thus bridges micro and mesoscale synaptic effects with network dynamics and behavior.

## Introduction

Episodic and semantic memory were originally proposed as distinct systems that compete in retrieval (Tulving, 1972). More recent studies suggest, however, that this division is rather vague (McCloskey and Santee, 1981; Howard and Kahana, 2002; Renoult et al., 2019), as neural correlates of episodic and semantic retrieval overlap (Weidemann et al., 2019). Episodic memory traces are susceptible to transformation and loss of information (Tulving, 1972), and this loss of episodicity can be attributed to semantization, which typically takes the form of a decontextualization process (Viard et al., 2007; Habermas et al., 2013; Duff et al., 2020). Baddeley (1988) hypothesized that semantic memory might represent the accumulated residue of multiple learning episodes, consisting of information which has been semanticized and detached from the associated episodic contextual detail. Extensions of the classical Remember/Know (R/K) behavioral experiment demonstrated that item-context decoupling can occur rapidly (Opitz, 2010). In these experiments, items were presented either in a unique context, or across several contexts. Low context variability improved the recollection rate, whereas context overload led to decontextualization and “Know” type of responses, i.e., recognition of item-only information without any detail about episodic context (Opitz, 2010; Smith and Manzano, 2010; Smith and Handy, 2014). To the best of our knowledge, there have not been any computational hypotheses proposed to offer mechanistic insights into this item-context decoupling effect.

Several computational spiking neural network models of cortical associative memory have previously been developed and used to investigate mechanisms underlying working memory maintenance and recall (Lundqvist et al., 2010, 2011; Herman et al., 2013). A similar model enhanced with a Bayesian–Hebbian learning rule (Bayesian Confidence Propagation Neural Network; BCPNN) representing synaptic and intrinsic plasticity was then used to study one-shot memory encoding (Fiebig and Lansner, 2017), and more recently, it was extended into a multinetwork cortical model to examine a novel “indexing theory” of working memory (Fiebig et al., 2020).

In the present study, relying on a similar spiking neural network model with identical modular architecture we propose and evaluate a Bayesian–Hebbian hypothesis about synaptic and network mechanisms underlying memory semantization and qualitatively match model output to available behavioral data. We show that associative binding between items and contexts becomes weaker when an item is presented across multiple contexts (high context variability). This gradual trace transformation relies on the nature of Bayesian learning, which normalizes and updates weights over estimated presynaptic (Bayesian-prior) as well as postsynaptic (Bayesian-posterior) spiking activity. We compare these findings with an analogous model that features the more well-known spike-timing-dependent plasticity (STDP) instead of the BCPNN learning rule, and demonstrate that no memory semantization effect can be reproduced, regardless of the degree of context variability. Notably, there have been earlier modeling attempts at semantization using STDP or other learning rules but this memory phenomenon has been interpreted differently involving slow memory consolidation (requiring sleep, repeated exposures, or systems consolidation) or extraction of semantic relations (a.k.a. prototype learning) among a group of episodic memories sharing statistical similarities (Deperrois et al., 2021; Remme et al., 2021). We argue that our hypothesis is more generic as it does not assume any statistical structure of the memory object representations. Finally, we also show how selective plasticity neuromodulation of one-shot learning (tentatively modeling effects of attention, emotional salience, and surprise on plasticity) may delay or prevent decontextualization.

In contrast to existing computational models of episodic memory (Norman and O’Reilly, 2003; Wixted, 2007; Greve et al., 2010), our model bridges behavioral outcomes with neural and synaptic mechanisms. It reproduces episodic memory phenomena on behavioral time scales under constrained network connectivity with plausible postsynaptic potentials, firing rates, and other biological parameters.

## Materials and Methods

### Neuron and synapse model

We use adaptive exponential integrate-and-fire point model neurons, which feature spike frequency adaptation, enriching neural dynamics and spike patterns, especially for the pyramidal cells (Brette and Gerstner, 2005). The neuron model is an effective model of cortical neuronal activity, reproducing a wide variety of electrophysiological properties, and offers a good phenomenological description of typical neural firing behavior, but it is limited in predicting the precise time course of the subthreshold membrane voltage during and after a spike or the underlying biophysical causes of electrical activity (Gerstner and Naud, 2009). We slightly modified it for compatibility with the BCPNN synapse model (Tully et al., 2014) by integrating an intrinsic excitability current.

Development of the membrane potential *V_m_* and the adaptation current *I_w_* is described by the following equations:

(1) $$C_{\text{m}}\frac{dV_{\text{m}}}{dt}=-g_{\text{L}}(V_{\text{m}}-E_{\text{L}})+g_{\text{L}}\Delta \tau e^{\frac{V_{\text{m}}-V_{\text{t}}}{\Delta \tau }}-I_{\text{w}}+I_{\text{ext}}+I_{\text{syn}}$$

(2) $$\frac{dI_{\text{w}}}{dt}=\frac{-I_{\text{w}}}{\tau_{I_{w}}}+b\delta (t-t_{\text{sp}}).$$

Equation 1 describes the dynamics of the membrane potential *V_m_* including an exponential voltage dependent activation term. A leak current is driven by the leak reversal potential *E_L_* through the conductance *g_L_* over the neural surface with a capacity *C_m_*. Additionally, *V_t_* is the spiking threshold, and Δ_T_ shapes the spike slope factor. After spike generation, membrane potential is reset to *V_r_*. Spike emission upregulates the adaptation current by *b*, which recovers with time constant $\tau_{I_{w}}$ (Table 1). To simplify the model, we have removed subthreshold adaptation, which is part of some AdEx models.

**Table 1:** Neuron model and synaptic parameters

Neuron model parameter	Symbol	Value	BCPNN parameter	Symbol	Value
Adaptation current	b	86 pA	BCPNN AMPA gain	$w_{gain}^{AMPA}$	0.76 nS
Adaptation decay time constant	$\tau_{I_{w}}$	280 ms	BCPNN NMDA gain	$w_{gain}^{NMDA}$	0.07 nS
Membrane capacitance	*C*_m_	280 pF	BCPNN bias current gain	*β*_gain_	40 pA
Leak reversal potential	*E*_L_	−70.6 mV	BCPNN lowest spiking rate	*f*_min_	0.2 Hz
Leak conductance	*g*_L_	14 nS	BCPNN highest spiking rate	*f*_max_	25 Hz
Upstroke slope factor	Δ_T_	3 mV	BCPNN lowest probability	*ϵ*	0.0026
Spike threshold	*V_t_*	−55 mV	P trace time constant	*τ*_p_	15 s
Spike reset potential	*V_r_*	−60 mV	Regular plasticity	*κ_normal_*	1
Refractory period	*τ*_ref_	5 ms	Modulated plasticity	*κ_boost_*	2
Receptor parameter	Symbol	Value	Short-term plasticity parameter	Symbol	Value
AMPA synaptic time constant	*τ* ^AMPA^	5 ms	Utilization factor	*U*	0.2
NMDA synaptic time constant	*τ* ^NMDA^	100 ms	Augmentation decay time constant	*τ*_A_	5 s
GABA synaptic time constant	*τ* ^GABA^	5 ms	Depression decay time constant	*τ*_D_	280 ms
AMPA reversal potential	*E* ^AMPA^	0 mV			
NMDA reversal potential	*E* ^NMDA^	0 mV			
GABA reversal potential	*E* ^GABA^	−75 mV			

Besides a specific external input current *I_ext_*, model neurons receive synaptic currents $I_{syn_{j}}$ from conductance based glutamatergic and GABAergic synapses. Glutamatergic synapses feature both AMPA/NMDA receptor gated channels with fast and slow conductance decay dynamic, respectively. Current contributions for synapses are described as follows:

(3) $$I_{\text{syn }j}=\sum_{syn}\sum_{i}g_{ij}^{syn}(t)(V_{\text{m}}-E_{ij}^{syn})=I_{j}^{AMPA}(t)+I_{j}^{NMDA}(t)+I_{j}^{GABA}(t).$$

The glutamatergic synapses are also subject to synaptic depression and augmentation with a decay factor *τ_D_* and *τ_A_*, respectively (Table 1), following the Tsodyks–Markram formalism (Tsodyks and Markram, 1997). We have chosen those time-constants from the plausible range of computational fits made on the basis of electrophysiological recordings of cortical pyramidal cells (Wang et al., 2006). The utilization factor u represents the fraction of available resources used up by each transmitted spike (a proxy of synaptic release probability), whereas x tracks the fraction of resources that remain available because of transmitter depletion (synaptic depression):

(4) $$\frac{du_{ij}^{}}{dt}=-\frac{u_{ij}^{}}{\tau_{A}}+U{\left(1-u_{ij}\right)}^{}\sum_{sp}\delta (t-t_{sp}^{i}-t_{ij})$$

(5) $$\frac{dx_{ij}^{}}{dt}=\frac{1-x_{ij}^{}}{\tau_{D}}-Ux_{ij}^{}\sum_{sp}\delta (t-t_{sp}^{i}-t_{ij}).$$

### Spike-based BCPNN plasticity

We implement synaptic plasticity of AMPA and NMDA connection components using the BCPNN learning rule (Lansner and Ekeberg, 1989; Wahlgren and Lansner, 2001; Tully et al., 2014). BCPNN is derived from Bayes rule, assuming a postsynaptic neuron employs some form of probabilistic inference to decide whether to emit a spike or not. Despite that it accounts for the basic Bayesian inference, it is considered more complex than the standard STDP learning rule (Caporale and Dan, 2008), and as such, it reproduces the main features of STDP plasticity.

The BCPNN synapse continuously updates three synaptic biophysically plausible local memory traces, *P_i_*, *P_j_*, and *P_ij_*, implemented as exponentially moving averages (EMAs) of preactivation, postactivation, and coactivation, from which the Bayesian bias and weights are calculated. EMAs prioritize recent patterns, so that newly learned patterns gradually replace old memories. Specifically, learning implements exponential filters, Z, and P, of spiking activity with a hierarchy of time constants, *τ_z_*, and *τ_p_*, respectively [the full BCPNN model implements additional eligibility E traces (Tully et al., 2014), which are not used here]. Because of their temporal integrative nature, they are referred to as synaptic (local memory) traces.

To begin with, BCPNN receives a binary sequence of presynaptic and postsynaptic spiking events (*S_i_*, *S_j_*) to calculate the traces *Z_i_* and *Z_j_*:

(6) $$\{\begin{array}{l} \tau_{{\text{z}}_{\text{i}}}\frac{dZ\text{i}}{dt}=\frac{S\text{i}}{f_{\text{max}}t_{\text{spike}}}-Z_{\text{i}}+\epsilon \\ \tau_{{\text{z}}_{\text{j}}}\frac{dZ_{\text{j}}}{dt}=\frac{S_{\text{j}}}{f_{\text{max}}t_{\text{spike}}}-Z_{\text{j}}+\epsilon \end{array},$$

*f_max_* denotes the maximal neuronal spike rate, *ϵ* is the lowest attainable probability estimate, *t_spike_* denotes the spike duration while $\tau_{{\text{z}}_{\text{i}}}=\tau_{{\text{z}}_{\text{j}}}$ are the presynaptic and postsynaptic time constants, respectively ( $\tau_{\text{z}}=\tau^{\text{AMPA}}=5$ ms for AMPA, and $\tau_{\text{z}}=\tau^{\text{NMDA}}=100$ ms for NMDA components; Table 1).

P traces are then estimated from the Z traces as follows:

(7) $$\{\begin{array}{l} \tau_{\text{p}}\frac{dP_{\text{i}}}{dt}=\kappa (Z_{\text{i}}-P_{\text{i}})\\ \tau_{\text{p}}\frac{dP_{\text{j}}}{dt}=\kappa (Z_{\text{j}}-P_{\text{j}})\\ \tau_{\text{p}}\frac{dP_{\text{ij}}}{dt}=\kappa (Z_{\text{i}}Z_{\text{j}}-P_{\text{ij}}) \end{array}.$$

The parameter *κ* adjusts the learning rate, reflecting the action of endogenous modulators of learning efficacy (i.e., activation of a D1R-like receptor). Setting *κ* = 0 freezes the network’s weights and biases, though in our simulations the learning rate remains constant (*κ* = 1) during encoding (see Results, Semantization of episodic representations in the BCPNN model and Item-context interactions under STDP). However, we trigger a transient increase of plasticity in specific scenarios to model preferential retention of salient events (see Results, Preferential retention; Table 1).

Finally, *P_i_*, *P_j_*, and *P_ij_* are used to calculate intrinsic excitability *β_j_* and synaptic weights *w_ij_* with a scaling factor *β_gain_* and $w_{gain}^{syn}$ respectively (Table 1):

(8) $$\{\begin{array}{l} w_{\text{ij}}=w_{gain}^{syn}log\frac{P_{\text{ij}}}{P_{\text{i}}P_{\text{j}}}\\ \beta_{\text{j}}=\beta_{\text{gain}}log(P_{\text{j}}) \end{array}.$$

### Spike-based STDP learning rule

In our study, we examine the impact on semantization when the STDP learning rule replaces BCPNN associative connectivity in the same episodic memory task. Synapses under STDP are developed and modified by a repeated pairing of presynaptic and postsynaptic spiking activity, while their relative time window shapes the degree of modification (Ren et al., 2010). The amount of trace modification depends on the temporal difference (Δ*_t_*) between the time point of the presynaptic action potential (*t_i_*) and the occurrence of the postsynaptic spike (*t_j_*) incorporating a corresponding transmission delay (*τ_d_*) from neuron *i* to neuron *j*:

(9) $$\Delta t=t_{j}-\left(t_{i}+\tau_{d}\right).$$

After processing Δ*t*, STDP updates weights accordingly:

(10) $$\Delta w_{ij}(\Delta t)=\{\begin{array}{ll} \lambda {\left(1-w\right)}^{\mu_{+}}e^{\left(-|\Delta t|/\tau_{+}\right)} & \text{if }\Delta t\geq \tau_{d}\\ \lambda \alpha w^{\mu_{-}}e^{\left(-|\Delta t|/\tau_{-}\right)} & \text{if }\Delta t<\tau_{d} \end{array}.$$

Here, *λ* corresponds to the learning rate, *α* reflects a possible asymmetry between the scale of potentiation and depression, *τ*_±_ control the width of the time window, while *μ*_±_ ∈ {0,1} allows to choose between different versions of STDP (i.e., additive, multiplicative; Morrison et al., 2008). Synapses are potentiated if the synaptic event precedes the postsynaptic spike and get depressed if the synaptic event follows the postsynaptic spike (Van Rossum et al., 2000).

Associative weights *w_ij_* are initialized to *w*_0_, and their maximum allowed values are constrained according to *w_max_* to ensure that synaptic weights are always positive and between [ $w_{0},w_{max}$] (Table 2). The resulting associative weight distributions are generally comparable in strength to the BCPNN model weights, but to make them match, we adjust *w_max_* in conjunction with a reasonably small learning rate *λ*. The maximum allowed weight (*w_max_*) is a necessary standard parameter of the default STDP we use in NEST (see below, Code accessibility). To obtain a stable competitive synaptic modification, the integral of Δ*w_ij_* must be negative (Song et al., 2000). To ensure this, we choose *α* = 1.2, which introduces an asymmetry between the scale of potentiation and depression along with a symmetric time window resulting in a ratio of $\alpha \tau_{-}/\tau_{+}$>1.0 (Ren et al., 2010). We set *μ*_±_ = 1 resulting in multiplicative STDP (in-between values lead to rules which have an intermediate dependence on the synaptic strength).

**Table 2:** STDP model parameters

Parameter	Symbol	Value
Weight initialization	*w*_0_	0 nS
AMPA maximum allowed weight	$w_{max}^{AMPA}$	13.5 nS
NMDA maximum allowed weight	$w_{max}^{NMDA}$	3.5 nS
Learning rate	*λ*	0.01
Asymmetry parameter	*α*	1.2
Weight dependence exponent, potentiation	*μ*_+_	1
Weight dependence exponent, depression	*μ*_–_	1
Symmetric time window	*τ*_±_	20 ms

**Table 3:** Network layout, connectivity, and stimulation protocol

Layout	Symbol	Value	Connectivity	Symbol	Value	Stimulation	Symbol	Value
Cortical patch size	*C_ps_*	2.0 × 1.5 mm	Axonal conduction speed	V	0.2 m/s	Background noise PYR (encoding)	$r_{bg-encoding}^{PYR}$	650 Hz
Simulated HCs (each network)	*n_HC_*	12	Myelinated axonal speed	*V_myel_*	2 m/s	Background noise PYR (recall)	$r_{bg-recall}^{PYR}$	450 Hz
Simulated MCs (each network)	*n_MC_*	120	Minimal synaptic delay	$t_{min}^{syn}$	1.5 ms	Background noise BA	$r_{bg}^{BA}$	75 Hz
Simulated MCs per HC	$n_{MC}^{HC}$	10	HC diameter	*d_HC_*	0.5 mm	Background conductance	$g_{bg}^{PYR,BA}$	± 1.5 nS
Number of items	*n_ITEM_*	4 (from 10)	Distance between networks	$d_{CONTEXT}^{ITEM}$	10 mm	Stimulation duration	*t_stim_*	250 ms
Number of contexts	*n_CONTEXT_*	10 (from 10)	PYR-PYR recurrent cp	*cp_PP_*	0.2	Stimulation rate	*r_stim_*	500 Hz
Layer 2/3 pyramidal per MC	$n_{MC}^{PYR-L23}$	30	PYR-PYR long-range cp	*cp_PPL_*	0.25	Cue stimulation length	*t_cue_*	50 ms
Basket cells per MC	$n_{MC}^{Basket}$	2	PYR-PYR associative cp	*cp_PPA_*	0.02	Cue stimulation rate	*r_cue_*	400 Hz
MC grid size (Item + Context)	$G_{MC}^{TOTAL}$	24 × 10	PYR-BA cp, BA-PYR cp	*cp_PB_*, *cp_BP_*	0.7	Stimulation and cue conductance	*g_stim_*	+1.5 nS
			PYR-BA cc	*g_PB_*	3 nS	Interstimulus interval	T*_stim_*	500 ms
			BA-PYR cc	*g_BP_*	−7 nS	Attractor detection threshold	*r_th_*	10 Hz

PYR, pyramidal cell; BA, basket cell; cp, connection probability; cc, connection conductance.

### Two-network architecture and connectivity

**Figure 1.**
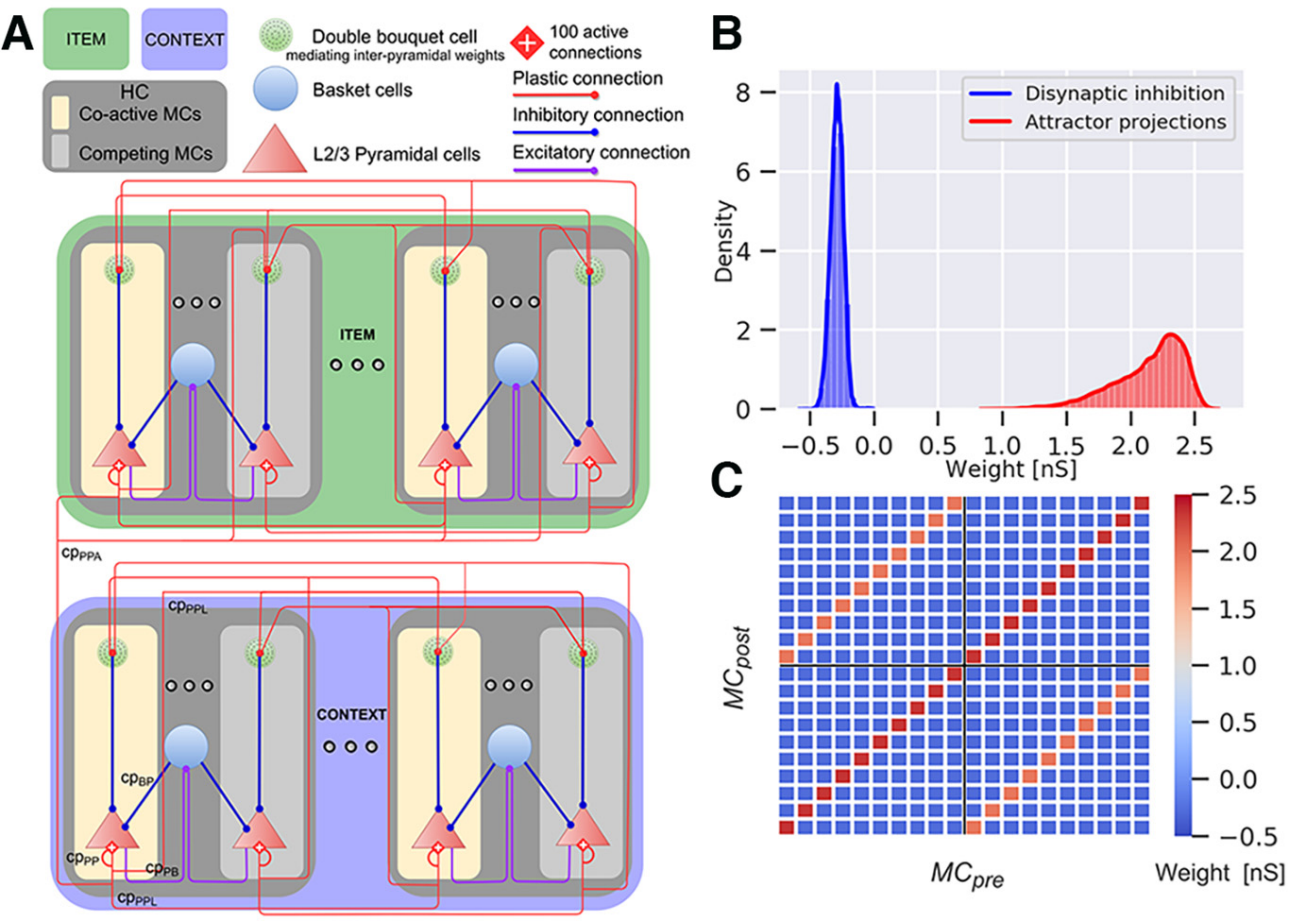
Network architecture and connectivity of the Item (green) and Context (blue) networks. ***A***, The model represents a subsampled modular cortical layer 2/3 patch consisting of MCs nested in HCs. Both networks contain 12 HCs, each comprising 10 MCs. We preload abstract long-term memories of item and context representations into the respective network, in the form of distributed cell assemblies with weights establishing corresponding attractors. Associative plastic connections bind items with contexts. The network features lateral inhibition via basket cells (purple and blue lines) resulting in a soft winner-take-all dynamics. Competition between attractor memories arises from this local feedback inhibition together with disynaptic inhibition between HCs. ***B***, Weight distribution of plastic synapses targeting pyramidal cells. The attractor projection distribution is positive with a mean of 2.1, and the disynaptic inhibition is negative with a mean of −0.3 (we show the fast AMPA weight components here, but the simulation also includes slower NMDA weight components). ***C***, Weight matrix between attractors and competing MCs across two sampled HCs. The matrix displays the mean of the weight distribution between a presynaptic (*MC_pre_*) and postsynaptic MC (*MC_post_*), within the same or different HC (black cross separates grid into blocks of HCs, only two of which are shown here). Recurrent attractor connections within the same HC are stronger (main diagonal, dark red) compared with attractor connections between HCs (off-diagonals, orange). Negative pyramidal-pyramidal weights (blue) between competing MCs amounts to disynaptic inhibition mediated by double bouquet cells.

The network model features two reciprocally connected networks, the so-called Item and Context networks. For simplicity, we assume that Item and Context networks are located at a substantial distance accounting for the reduced internetwork connection probabilities (Table 3). Each network follows a cortical architecture with modular structure compatible with previous spiking implementations of attractor memory networks (Lansner, 2009; Lundqvist et al., 2011; Tully et al., 2014, 2016; Fiebig and Lansner, 2017; Chrysanthidis et al., 2019; Fiebig et al., 2020), and is best understood as a subsampled cortical layer 2/3 patch with nested hypercolumns (HCs) and minicolumns (MCs; Fig. 1*A*). Both networks span a regular-spaced grid of 12 HCs (Table 3), each with a diameter of 500 μm (Mountcastle, 1997). In our model, items are embedded in the Item network and context information in the Context network as internal well consolidated long-term memory representations (cell assemblies), supported via intranetwork weights derived using prior BCPNN learning with long time constant (Fig. 1*B*,*C*; Table 3). Consequently, these weights were resistant to changes during associative learning of projections between Item and Context networks (see Results). Our item and context memory representations are distributed and nonoverlapping, i.e., with a single distinct pattern-specific (encoding) MC per HC. This results in sparse neocortical activity patterns (Barth and Poulet, 2012). It should be noted that the model tolerates only a marginal overlap between different memory patterns, i.e., shared encoding MCs (data not shown). Each MC is composed of 30 pyramidal cells (representing the extent of layer 2/3) with shared selectivity, forming a functional (not strictly anatomic) column. In total, the 24 HCs (10 MCs each) of the model contain 7200 excitatory and 480 inhibitory cells, significantly downsampling the number of MCs per HC (∼100 MCs per HC in biological cortex). The high degree of recurrent connectivity within (Thomson et al., 2002; Yoshimura and Callaway, 2005) and between MCs links coactive MCs into larger cell assemblies (Stettler et al., 2002; Binzegger et al., 2009; Muir et al., 2011; Eyal et al., 2018). Long-range bidirectional internetwork connections (item-context bindings or associative connections) are plastic (shown in Fig. 1*A* only for MC1 in HC1 of the Context network), binding items, and contextual information (Ranganath, 2010). On average, recurrent connectivity establishes 100 active plastic synapses onto each pyramidal cell from other pyramidals with the same selectivity, because of a sparse internetwork connectivity (*cp_PPA_*) and denser local connectivity (*cp_PP_*, *cp_PPL_*; connection probability refers to the probability that there is a connection between a randomly selected pair of neurons from given populations; in Fig. 1*A*, connection probabilities are only shown for MC1 in HC1 of the Context network). The model yields biologically plausible EPSPs for connections within HCs (0.45 ± 0.13 mV), measured at resting potential *E_L_* (Thomson et al., 2002). Densely recurrent nonspecific monosynaptic feedback inhibition mediated by fast spiking inhibitory cells (Kirkcaldie, 2012) implements a local winner-take-all structure (Binzegger et al., 2009) among the functional columns. IPSPs have an amplitude of −1.160 mV (±0.003) measured at −60 mV (Thomson et al., 2002). These bidirectional connections between basket and pyramidal cells within the local HCs are drawn with a 70% connection probability. Notably, double bouquet cells shown in Figure 1*A* are not explicitly simulated, but their effect is nonetheless expressed by the BCPNN rule. A recent study based on a similar single-network architecture (i.e., with the same modular organization, microcircuitry, conductance-based AdEx neuron model, cell count per MC and HC) demonstrated that learned mono-synaptic inhibition between competing attractors is functionally equivalent to the disynaptic inhibition mediated by double bouquet and basket cells (Chrysanthidis et al., 2019). Parameters characterizing other neural and synaptic properties including BCPNN can be found in Table 1.

Figure 1*B* shows the weight distributions of embedded distributed cell assemblies, representing different memories stored in the Item and Context networks. Attractor projections can be further categorized into strong local recurrent connectivity within HCs, and slightly weaker long-range excitatory projections across HCs (Fig. 1*C*).

### Axonal conduction delays

Conduction delays (*t_ij_*) between a presynaptic neuron *i* and a postsynaptic neuron *j* are calculated based on their Euclidean distance, *d*, and a conduction velocity *V* (Eq. 11). Delays are randomly drawn from a normal distribution with a mean according to distance and conduction velocity, with a relative SD of 30% of the mean to account for individual arborization differences, and varying conduction speed as a result of axonal thickness and myelination. In addition, a minimal delay of 1.5 ms ( $t_{min}^{syn}$; Table 3) is added to reflect synaptic delays because of effects that are not explicitly modelled, e.g., diffusion of neurotransmitters over the synaptic cleft, dendritic branching, thickness of the cortical sheet and the spatial extent of columns (Thomson et al., 2002). Associative internetwork projections have a 10-fold faster conduction speed than those within each network, reflecting axonal myelination:

(11) $$\bar{t_{ij}}=\frac{d}{V}+t_{min}^{syn},\;t_{ij}∼N\left(\bar{t_{ij}},\;.3\;\bar{t_{ij}}\right).$$

### Stimulation protocol

Noise input to pyramidal cells and fast spiking inhibitory basket cells is a zero-mean noise, generated by two independent Poisson generators with opposing driving potentials. Pyramidal cells coding for specific items and contexts are stimulated with an additional specific excitation during encoding and cued recall (all parameters in Table 3). Item-context association encoding is preceded by a brief period of background noise excitation to avoid initialization transients.

### Attractor activation detector

We detect and report cued recall of items or contexts by using an attractor activation detection algorithm based on EMAs of spiking activity. Pattern-wise EMAs are calculated using Equation 12, where the δ function *δ* denotes the spike events of a pattern-selective neural population of *n_pop_* = 30 pyramidal cells. The filter time constant *τ* = 40 ms is much larger than the sampling time interval Δ*T *=* *1 ms:

(12) $$e_{0}=0,e_{t}=\frac{\Delta T}{\tau }e_{t-\Delta T}+\delta_{t}\frac{1}{\tau n_{pop}}.$$

Pattern activations are detected by a simple threshold (*r_th_*) at about 10-fold the baseline activity with a small caveat: to avoid premature offset detection because of synchrony in fast spiking activity, we only count activations as terminated if they do not cross the threshold again in the next 40 ms. This method is highly robust because of the explosive dynamics of recurrent spiking activity for activated attractors in the network. Any attractor activation that crosses this threshold for at least 40 ms is considered a successful recall.

### Code accessibility

We use Neural Simulation Tool (NEST) version 2.2.2, and a custom-built BCPNN learning rule module (Tully et al., 2014) running on a Cray XC-40 Supercomputer. NEST simulates the dynamics of spiking neural models and features a convenient Python interface (PyNEST) to NEST’s simulation kernel (Gewaltig and Diesmann, 2007). The custom-built spiking neural network implementation of the BCPNN learning rule for message passing interface (MPI) parallelized NEST is freely available online at Zenodo (https://doi.org/10.5281/zenodo.5101626) and also on Github using a Singularity container platform (https://github.com/Nikolaos-Chrysanthidis/BCPNN-NEST2.2.2-MPICH-SINGULARITY). The custom NEST BCPNN module is available as Extended Data 1. Further, the model is also available on ModelDB (https://modeldb.yale.edu/257610).

10.1523/ENEURO.0062-22.2022.ed1 Extended Data 1 BCPNN_NEST_Module. Download Extended Data 1, ZIP file.

## Results

### Semantization of episodic representations in the BCPNN model

**Figure 2.**
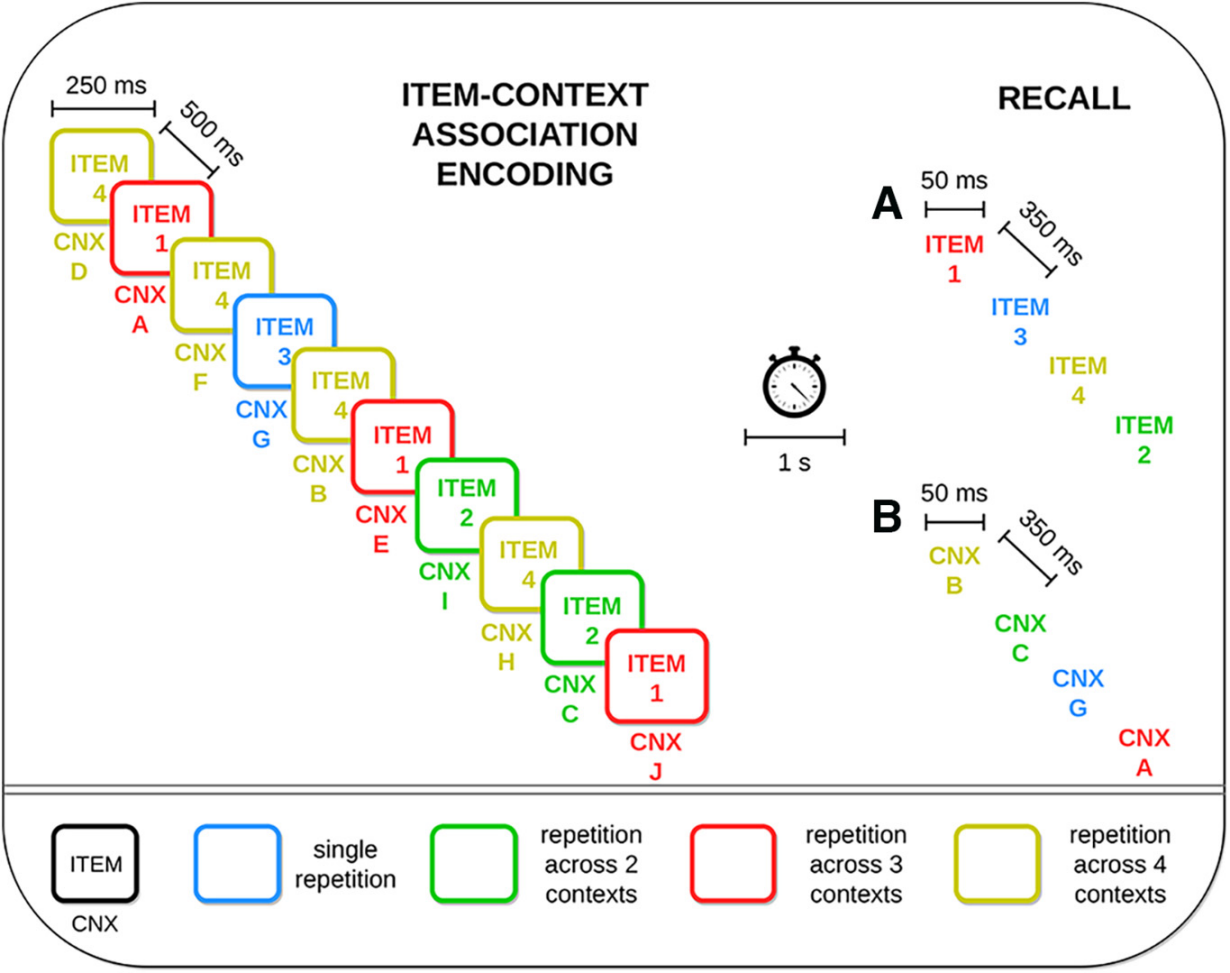
Trial structure of the two simulated variants of the episodic memory task. Items are first associated with one or several contexts (CNX) during the encoding phase in 250-ms cue episodes, with an interstimulus interval of 500 ms. The colors of the coactivated contexts are consistent with their corresponding associated item. The recall phase occurs with a delay of 1 s and involves different trials with either brief cues (50 ms) of the (***A***) items or (***B***) contexts presented during the item-context association encoding phase.

**Figure 3.**
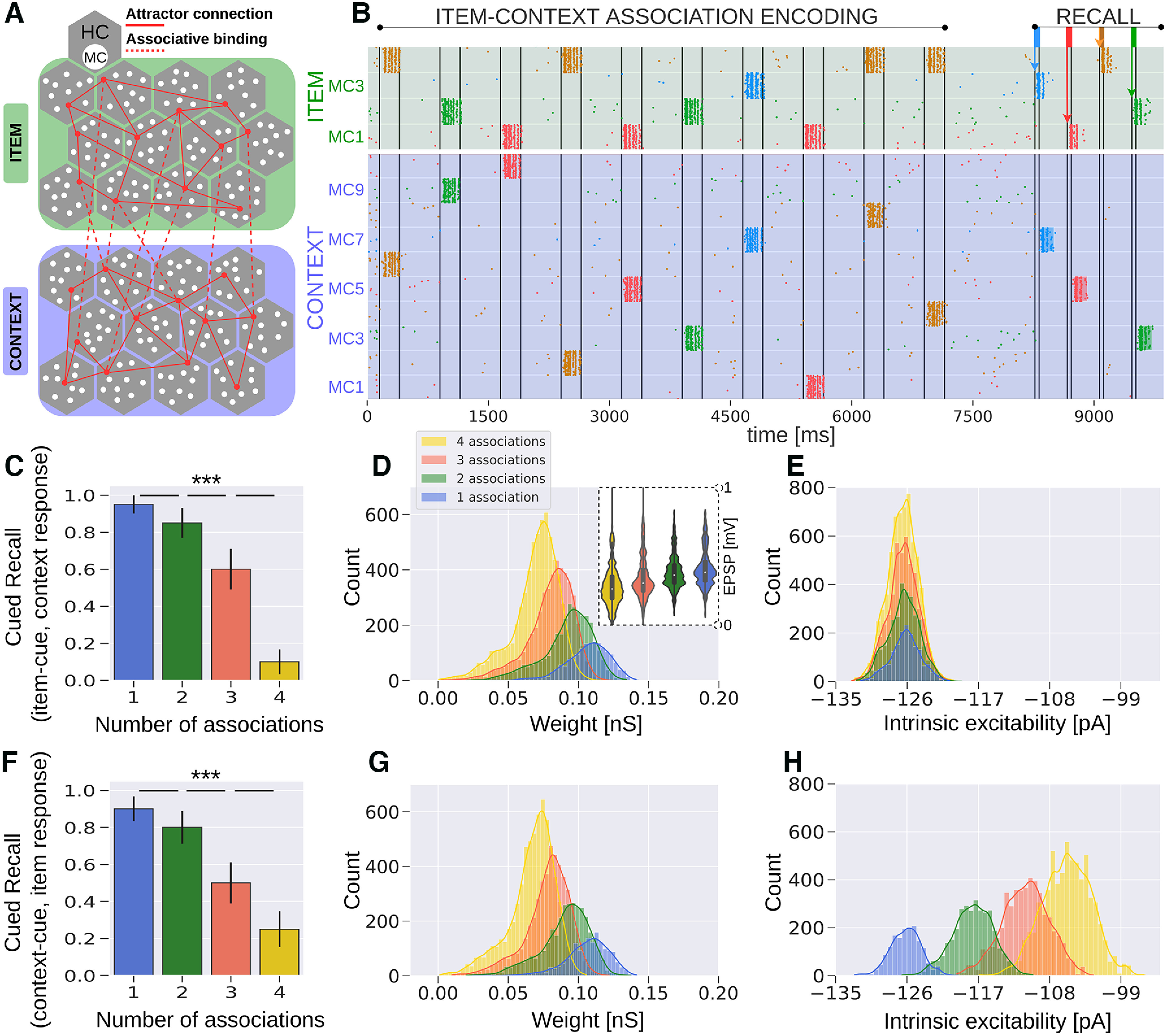
Semantization of episodic memory traces. ***A***, Schematic of the Item (green) and Context (blue) networks. Attractor projections are long-range connections across HCs in the same network and learned associative projections are connections between networks. ***B***, Spike raster of pyramidal neurons in HC1 of both the Item and Context networks. Each context/item memory pattern corresponds to the activation of a unique set of MCs in its network. Items and their corresponding context representations are simultaneously cued in their respective networks (compare Fig. 2*A*). Each item is drawn with a unique color, while contexts inherit their coactivated item’s color in the raster (i.e., the yellow pattern in the Item network is repeated over four different contexts, forming four separate associations marked with the same color). The testing phase occurs 1 s after the encoding. Brief 50-ms cues of already studied items trigger their activation. Following item activation, we detect evoked attractor activation in the Context network. ***C***, Average cued recall performance in the Context network (20 trials). The bar diagram reveals progressive loss of episodic context information (i.e., semantization) over the number of context associations made by individual cued items (compare Fig. 2*A*). ***D***, Distribution of plastic connection weights between the Item and Context networks (NMDA component shown here). Weights are noticeably weaker for items which participate in multiple associations. The distributions of synaptic weights exhibit a broader range for the items with multiple context associations, as the sample size is larger. The inset displays the distribution of EPSPs for the binding between Item and Context networks. The EPSP distributions follow the trend of the associative weights. The amplitudes (<1 mV) are lower for higher context variability. ***E***, The distribution of intrinsic excitability currents of pyramidal cells coding for specific context representations. The intrinsic excitability features similar distributions because each context is activated exactly once, regardless of whether the associated item forms multiple associations or not. ***F***, Average cued recall performance in the Item network (20 trials). Decontextualization over the number of associations is also observed when we briefly cue episodic contexts instead (compare Fig. 2*B*). ***G***, Distribution of strength of plastic connections from the contexts to their associated items. Analogously to ***D***, synapses weaken once an item is encoded in another context. ***H***, Intrinsic plasticity distribution of cells in the Item network. Intrinsic excitability distributions are higher for pyramidal cells coding for repeatedly activated items; ***p < 0.001 (Mann–Whitney, *N *=* *20 in ***C***, ***F***). Error bars in ***C***, ***F*** represent SDs of Bernoulli distributions. Distributions of one, two, three, and four associations in ***D*, *G*, *H*** show significant statistical difference (*p* < 0.001, Mann–Whitney, *N *=* *2000).

An episodic memory task simulated in this work is inspired by a seminal memory effect shown in an experimental study by Opitz (2010). We deliberately abstract away some details of Opitz (2010) experimental design to provide a qualitative proof of principle with as few task assumptions as possible. This approach also offers a more generalized computational framework for studying the interplay of synaptic learning and its outcomes. In the same spirit, the systems architecture of our model is reduced to the Item and Context networks storing item and context information, respectively, as internal long-term memory representations (Fig. 1; for details, see Two-network architecture and connectivity and Table 3). We stimulate some items in a single context and others in a few different contexts establishing multiple associations (Fig. 2). Stimulus duration during encoding is *t_stim_* = 250 ms with a T*_stim_* = 500 ms interstimulus interval, and a test phase occurs after a 1-s delay period, which contains brief *t_cue_* = 50-ms cues of previously learned items (Table 3). Figure 3*A* illustrates an item-context pair, established by an associative binding through plastic bidirectional BCPNN projections (dashed lines). Item and context attractors (solid red lines) are embedded in each network and remain fixed throughout the simulation, representing well-consolidated long-term memory. We show an exemplary spike raster of pyramidal neurons in HC1 of both the Item and Context networks reflecting a trial simulation (Fig. 3*B*). Herein, item-3 (blue) establishes a single association, while item-4 (yellow) is encoded in four different contexts (Figs. 2*A*, 3*B*). We find clear evidence for strong item-context decoupling. The yellow item-4 (but not the blue item-3) is successfully recognized when cued but without any corresponding accompanying activation in the Context network (Fig. 3*B*). Figure 3*C* demonstrates that this item-context decoupling effect holds true also for the multi trial average as the performance of contextual retrieval when items serve as cues deteriorates with a higher context overload. Successful item recognition without any contextual information retrieval accounts for a “Know” response, as opposed to “Remember” judgments, which are accompanied by context recall. In fact, episodic loss in our network implies that no context is recalled despite the item memory activation. To elucidate this observed progressive loss of episodicity with the higher context variability (Fig. 3*C*), we sample and analyze the learned weight distributions of item-context binding recorded after the association encoding period (Fig. 3*D*). The item-context weight distribution in the one-association case is significantly stronger than in the two-association, three-association, or four-association case (*p *<* *0.001, Mann–Whitney, *N *=* *2000). This progressive weakening of weights leads to significantly lower EPSPs for the associative projections (*p *<* *0.05 for one vs two associations; *p *<* *0.001 for two vs three and three vs four associations, Mann–Whitney, *N *=* *300; Fig. 3*D*, see inset). To measure EPSPs, we stimulate individually all the neurons in HC1 of an item which forms one, two, three, or four associations and record the postsynaptic potential onto their associated context neurons. EPSPs are in general below 1 mV (Thomson et al., 2002; Song et al., 2005), measured at resting potential *E_L_* (Table 1), after item-context association encoding phase. Therefore, we attribute the loss of episodicity to a statistically significant weakening of the associative weight distributions with the increasing number of associated contexts. The associative weight distributions shown here refer to the NMDA component, while the weight distributions of the faster AMPA receptor connections display a similar trend (data not shown). The gradual trace modification we observe is a product of Bayesian learning, which normalizes and updates weights over estimated presynaptic (prior) as well as postsynaptic (posterior) spiking activity (see below, BCPNN and STDP learning rule in a microcircuit model for details).

Our simulation results are in line with related behavioral studies (Opitz, 2010; Smith and Manzano, 2010; Smith and Handy, 2014), which also reported item-context decoupling as the items were presented across multiple contexts. In particular, Opitz (2010) concluded that repetition of an item across different contexts (i.e., high context variability) leads to item-context decoupling, which is in agreement with our study. Furthermore, Smith and Manzano (2010) demonstrated in an episodic context variability task configuration, that recall deteriorates with context overload (number of words per context). Mean recall drops from ∼0.65 (one word per context) to 0.50 (three words per context), reaching ∼0.33 in the most overloaded scenario (fifteen words per context).

In Figure 3*E*, we show the distribution of intrinsic excitability over units representing different contexts. Pyramidal neurons in the Context network have a similar intrinsic excitability, regardless of their selectivity because all the various contexts are encoded exactly once.

Next, analogously to the previous analysis, we show that item-context decoupling emerges also when we briefly cue contexts rather than items during recall testing (Fig. 2*B*). In agreement with experimental data (Smith and Manzano, 2010; Smith and Handy, 2014), we obtain evidence of semantization as items learned across several discrete contexts are hardly retrieved when one of their associated contexts serves as a cue (Fig. 3*F*). We further sample and present the underlying associative weight distribution, between the Context and the Item networks (Fig. 3*G*). The distributions again reflect the semantization effect in a significant weakening of the corresponding weights. In other words, an assembly of pyramidal neurons representing items encoded across multiple contexts receives weaker projections from the Context network. At about four or more associations, the item-context binding becomes so weak that it fails to deliver sufficient excitatory current to trigger associated representations in the Item network. At the same time, intrinsic excitability of item neurons increases with the number of associated contexts corresponding to how much these neurons were active during the encoding phase (Fig. 3*H*; cf. Egorov et al., 2002; Tully et al., 2014).

### Item-context interactions under STDP

In this section, we contrast the results obtained with the BCPNN synaptic learning rule with those deriving from the more commonly used STDP learning rule in the same episodic memory task (Fig. 2; Spike-based STDP learning rule). The modular network architecture as well as neural properties and embedded memory patterns remain identical, but associative projections between networks are now implemented using a standard STDP synaptic learning rule (Morrison et al., 2008). The parameters of the STDP model are summarized in Table 2.

**Figure 4.**
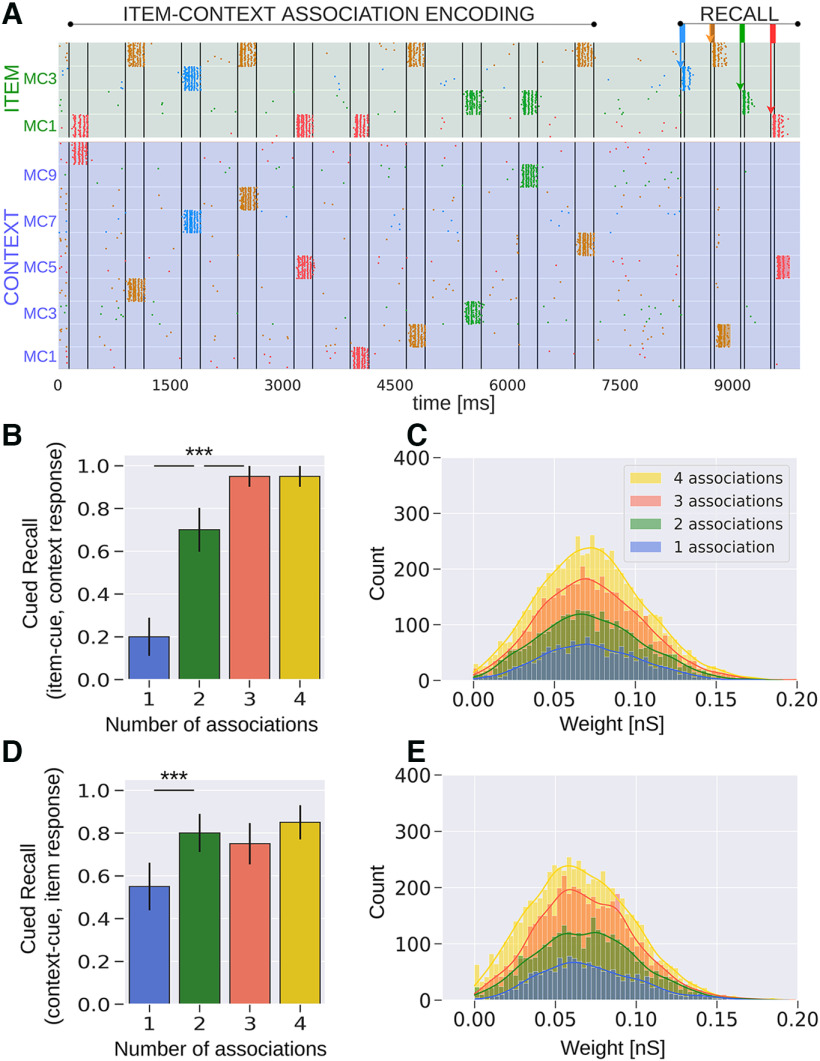
Network model where associative projections are implemented using standard STDP synaptic plasticity. ***A***, Spike raster of pyramidal neurons in HC1 of both the Item and Context networks. ***B***, Average item-cued recall performance in the Context network (20 trials). Episodic context retrieval is preserved even for high context variability (as opposed to BCPNN; compare Fig. 3*C*). ***C***, Distribution of NMDA receptor mediated synaptic weights between the item and context neural assemblies following associative binding. The distributions of item-context weights have comparable means at ∼0.065 nS regardless of how many context associations a given item forms. Bins merely display a higher count for the four-association case as the total count of associative weights is more extensive compared with items with fewer associations. ***D***, Average cued recall performance in the Item network when episodic contexts are cued (20 trials). ***E***, Distribution of NMDA component weights between associated context and item assemblies; ***p < 0.001 (Mann–Whitney, *N *=* *20 in ***B***, ***D***). Error bars in ***B***, ***D*** represent SDs of Bernoulli distributions.

Figure 4*A* shows an exemplary spike raster of pyramidal cells in HC1 of both the Item and the Context networks, based on the first variant of the episodic memory task described in Figure 2*A*. As earlier, items are encoded in a single or in multiple different contexts and they are briefly cued later during recall. A successful item activation may lead to a corresponding activation of its associated information in the Context network. We detect these activations as before (see Materials and Methods, Attractor activation detector) and report the cued recall score over the number of associations (Fig. 4*B*).

**Figure 5.**
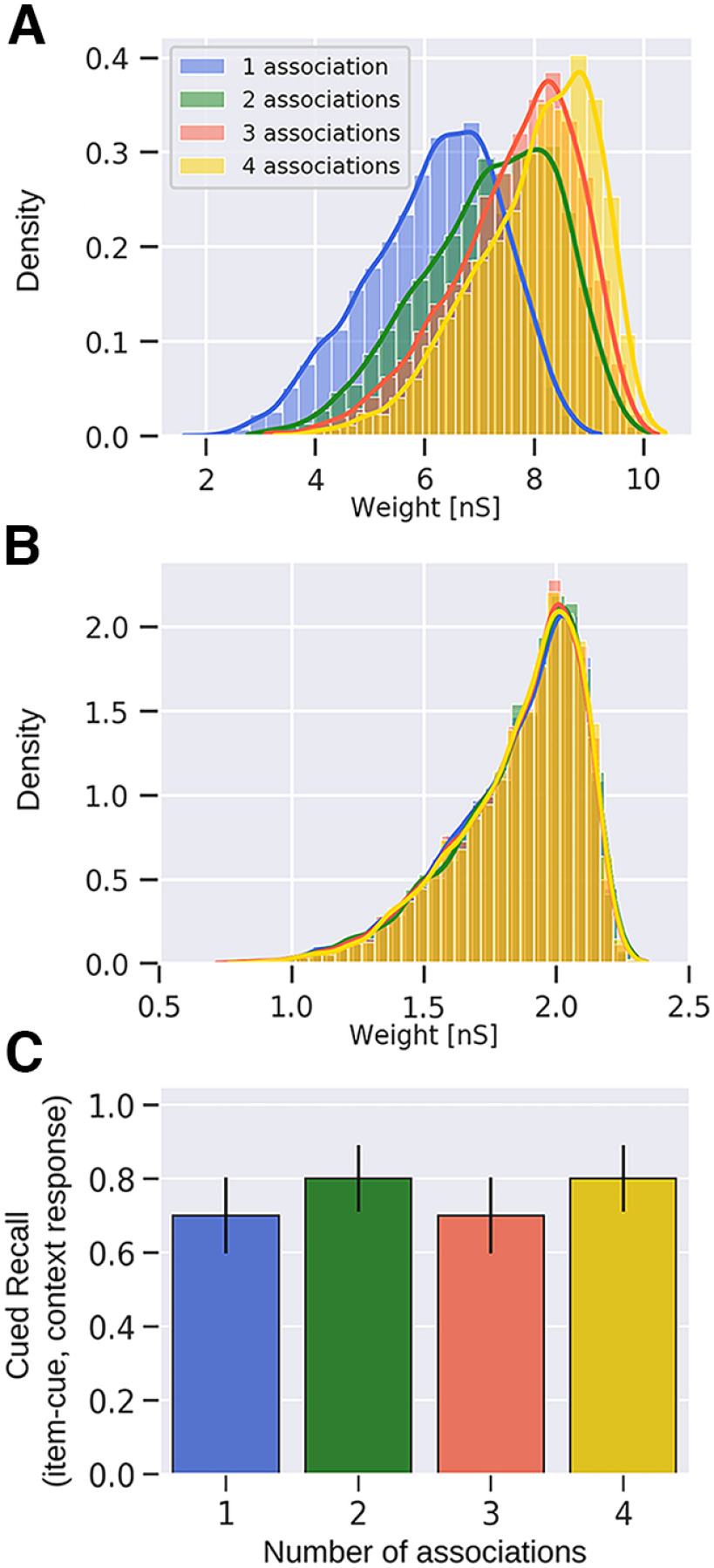
Removal of the augmentation mechanism in the network model. ***A***, Distribution of AMPA component weights of the Item network including synaptic augmentation. The multiplicative effect of synaptic augmentation on the consolidated items features stronger combined synaptic strength for items with higher context variability. Slower NMDA receptor weights follow a similar pattern. Weight distributions of one, two, three, and four associations have statistical difference (*p* < 0.001, Mann–Whitney, *N* = 2000). ***B***, Distribution of AMPA component weights of the Item network after removing synaptic augmentation. ***C***, Cued recall under STDP after removing synaptic augmentation. Average item-cued recall performance in the Context network (20 trials). To compensate for the removal of augmentation, we increased the stimulation rates and the synaptic gain eliciting comparable spiking activity. Error bars represent SDs of Bernoulli distributions.

Unlike the BCPNN network, we observe no evidence of semantization for high context variability. Instead, recollection is noticeably enhanced with an increase in the number of associations, which is in fact the opposite of what would be needed to explain item-context decoupling. STDP generates similarly strong associative binding regardless of context variability (Fig. 4*C*). The enhanced recollection in high context variability cases stems from the multiplicative effect of synaptic augmentation in the Tsodyks–Makram model on the Hebbian attractor weights. Items stimulated multiple times (e.g., four times) have a higher likelihood of being encoded near the end of the task, leading to more remaining augmentation during testing, thus, effectively boosting cued recall (Fig. 5*A*). This recency effect diminishes after removing synaptic augmentation from the model as attractor weights in the Item network have comparable distributions leading to similar cued recall performance regardless of context variability (Fig. 5*B*,*C*). As far as the context-cued variant of the task is concerned (Fig. 2*B*), there are also no signs of item-context decoupling for high context variability (Fig. 4*D*). The associative projections between Context and Item networks again have distributions with comparable means over context variability (Fig. 4*E*). Overall, decontextualization is not evident in either variant of the episodic memory task under the STDP learning rule.

### BCPNN and STDP learning rules in a microcircuit model

To better elucidate the emergent synaptic changes of the BCPNN and STDP model, we also apply these learning rules in a highly reduced microcircuit of spiking neurons. To this end, we now track the synaptic weight changes continuously. The neural and synaptic parameters (and most importantly all the plasticity parameters) used for the highly reduced BCPNN and STDP model are identical to the ones used for the large scale BCPNN and STDP model, respectively (see Tables 1, 2).

**Figure 6.**
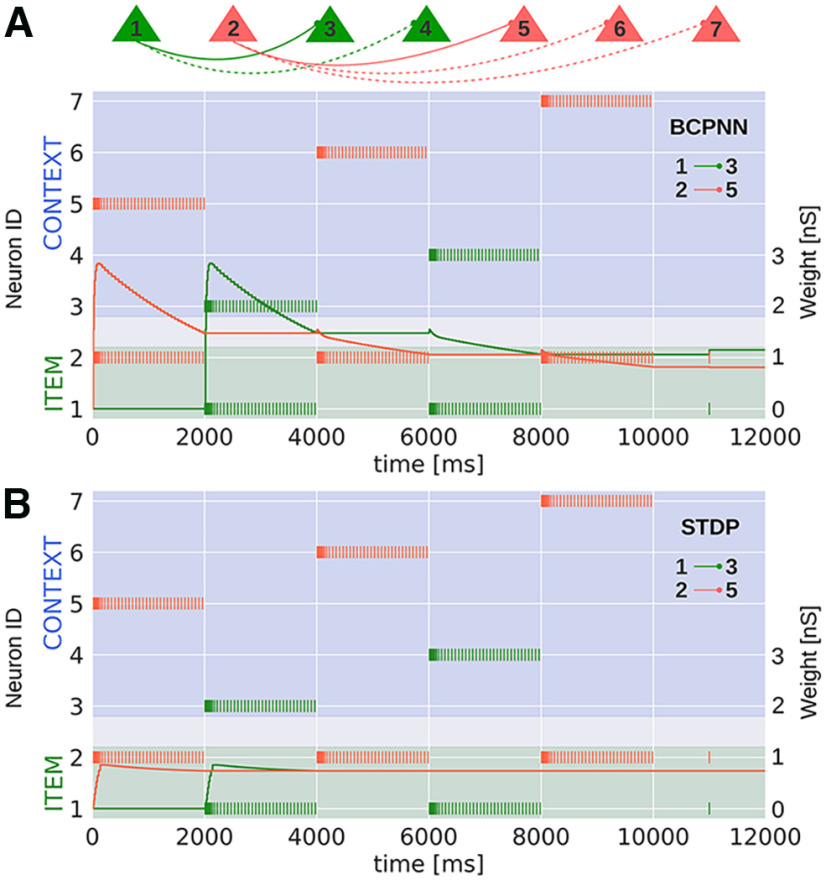
Continuous weight recordings in a microcircuit model with plastic synapses under the ***A***, BCPNN or ***B***, STDP learning rule. Neural and synaptic parameters correspond to those in the scaled model. In both cases, two item neurons (ID = 1,2) are trained to form two or three associations, respectively (dashed connections are simulated but their weight development is not shown here). During training, neurons are stimulated to fire at 20 Hz for 2 s. We display the developing synaptic weight between specific item-context pairs (ID = 1 and 3 in the 2-association scenario) and (ID = 2 and 5 in the 3-association scenario), and compare the converged weight values between the two-association and three-association case under both learning rules, following a final readout spike at 11 s.

First, we apply the BCPNN learning rule to the microcircuit model. We consider two separate item neurons (ID = 1 and 2), which form two or three associations with context neurons (ID = 3, 4, or 5, 6, 7), respectively (Fig. 6*A*). We display the synaptic strength development of the synapse between item neuron-1 and context neuron-3 (two associations, green), as well as the synapse between item neuron-2 and context neuron-5 (three associations, red) over the course of training these associations via targeted stimulation. BCPNN synapses get strengthened when the item-context pairs are simultaneously active and weaken when the item in question is activated with another context. Therefore, synapses of the item neuron that is encoded in three different contexts converge on weaker weights (Fig. 6*A*, 12 s), than those of the item neuron with two associated contexts. Weight modifications in the microcircuit model reflect the synaptic alterations observed in the large-scale network. BCPNN weights are shaped by traces of activation and coactivation (Eqs. 7, 8; Materials and Methods), which also get updated during the activation of an item within another context. For example, the item neuron-1 and context neuron-3 are not stimulated together between 6 and 8 s, but neuron-1 and context neuron-4 are. Thus, the P traces of the item activation (*P_i_*) increase, while the ones linked to context-3 (*P_j_*) decay with a time constant of 15 s (Table 1). Since the item and context neuron (ID = 1, 3) are not stimulated together, their coactivation traces (*P_ij_*) decay between 6 and 8 s. Overall, this leads to a weakening of the weight and hence to a gradual decoupling (Eq. 8; Materials and Methods).

In the same manner, we keep track of weight change in a microcircuit with the STDP learning rule (Fig. 6*B*). Unlike the microcircuit with BCPNN presented in Figure 6*A*, the STDP weights corresponding to the associations made by both item neurons converge to similar values, although they are associated with different number of contexts. As before, the synapse between an item neuron and an associated context neuron strengthens when this pair is simultaneously active, but remains stable when the item neuron is encoded in another context. For instance, the synapse between item neuron-2 and context neuron-5 strengthens when this pair is encoded (0–2 s), yet remains unaffected when item neuron-2 is activated in another context (i.e., context neuron-6, 4–6 s). This synaptic behavior explains the observed differences between the BCPNN and STDP large-scale model.

### Preferential retention

Several studies propose that one-shot salient events promote learning, and that these memories can be retained on multiple time scales ranging from seconds to years (Frankland et al., 2004; Petrican et al., 2010; Gruber et al., 2016; Panoz-Brown et al., 2016; Eichenbaum, 2017; Sun et al., 2018). Hypothetical mechanisms behind these effects are dopamine release and activation of DR1 like receptors, resulting in synapse-specific enhancement (Otmakhova and Lisman, 1996; Kuo et al., 2008), and systems consolidation (McClelland et al., 1995; Fiebig and Lansner, 2014). Overall, salient or reward driven events may be encoded more strongly as the result of a transient plasticity modulation. Recall from long-term memory is often viewed as a competitive process in which a memory retrieval does not depend only on its own synaptic strength but also on the strength of other components (Shiffrin, 1970). In view of this, we study the effects of plasticity modulation on encoding specific items within particular contexts, with the aim of investigating the role of enhanced learning for semantization in our model.

**Figure 7.**
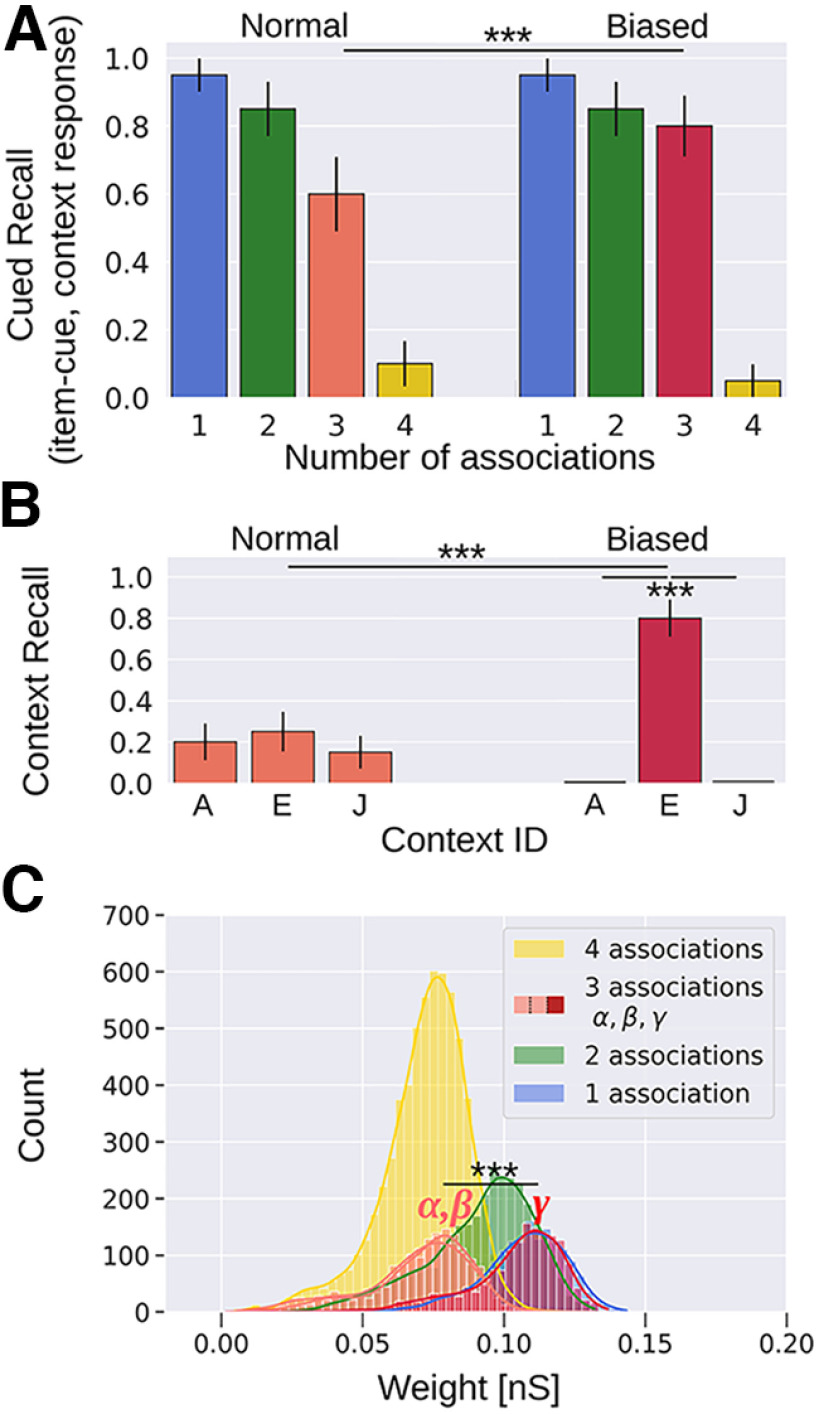
Plasticity modulation of a specific item-context pair enhances recollection and counteracts semantization. ***A***, Context recall performance. One of the pairs (context-E, item-1) presented in the episodic memory task (compare Fig. 2*A*) is subjected to enhanced plasticity during encoding, resulting in the boosted recall rate (3 associations, Normal vs Biased, 20 trial average). ***B***, Individual context retrieval contribution in the overall recall (3 associations). Retrieval is similar among the three contexts since plasticity modulation is balanced (left: Normal, *κ* = *κ_normal_*; compare Table 1). However, when context-E is encoded with enhanced learning (with item-1), its recall increases significantly (right: Biased, *κ* = *κ_boost_*; compare Table 1). ***C***, Weight distributions of the NMDA weight component. Encoding item-1 with context-E under modulated plasticity yields stronger synaptic weights [3 association, *α*,*β* (light red, highly overlapping distributions) vs *γ* (dark red)]; ***p < 0.001 (Mann–Whitney, *N *=* *20 in ***A***, ***B***, *N *=* *2000 in ***C***)]. Error bars in ***A***, ***B*** represent SDs of Bernoulli distributions. Weight distributions of one, two, three-*α*,-*β*, and four associations in ***C*** show significant statistical difference (*p* < 0.001, Mann–Whitney, *N *=* *2000).

Using the same network and episodic memory task as before (Fig. 2*A*), we modulate plasticity during the encoding of item-1 (red) in context-E via *κ* = *κ_boost_* (Eq. 7; Materials and Methods; Table 1). This results in an increased cued recall probability for the item associated with three episodic contexts relative to the unmodulated control (Fig. 7*A*, Normal vs Biased scenario, three associations). Episodic retrieval improves from 0.6 (Normal; Fig. 7*A*, left) to 0.8 (Biased, modulated plasticity; Fig. 7*A*, right) when item-1 is cued, which now performs more similarly to item with just two associated contexts. We further analyze and compare the recall of each context when its associated item-1 is cued (Fig. 7*B*, three associations). The control scenario (Normal; Fig. 7*B*, left) without transient plasticity modulation shows that the three contexts (ID = A, E, and J) are all recalled with similar probabilities. In contrast, encoding a specific pair with enhanced learning (upregulated *κ* = *κ_boost_*) yields higher recall for the corresponding context. In particular, the plasticity enhancement during associative encoding of the context-E (with item-1) results in an increased recall score to 0.8 (0.25 control), while the other associated contexts, ID = A and J, are suppressed (Fig. 7*B*), primarily because of soft winner-take-all competition between contexts (Fig. 1*A*).

We attribute these changes to the stronger weights because of enhanced learning (Fig. 7*C*, dark red distribution, *γ*). Weights between unmodulated item-context pairs (item-1 and context-A,-J) show mostly unaltered weight distributions (*α*,*β*, light red), while the biased associative weight distribution between item-1 and context-E is now comparable to the weight distribution of the one-association case. Performance does not exactly match that case though because of some remaining competition among the three contexts. Overall, these results demonstrate how a single salient episode may strengthen memory traces and thus impart resistance to semantization (Rodríguez et al., 2016).

## Discussion

The primary objective of this work was to explore the interaction between synaptic plasticity and context variability in the semantization process. To cast new light on the episodic-semantic interplay, we built a cortical memory model of two spiking neural networks that feature the same modular architecture. The networks are coupled with plastic associative connections, which collectively represent distributed cortical episodic memory. Our results suggest that some forms of plasticity offer a synaptic explanation for the cognitive phenomenon of semantization, thus bridging scales and linking network connectivity and dynamics with behavior. We use a spiking neuronal network model combined with BCPNN, which allows us to directly compare it with a standard Hebbian STDP learning rule. In particular, we demonstrated that with Bayesian–Hebbian (BCPNN) synaptic plasticity, but not with standard Hebbian STDP, the model can reproduce traces of semantization as a result of learning. Notably, this was achieved with biologically constrained network connectivity, postsynaptic potential amplitudes, and firing rates compatible with mesoscale recordings from cortex and earlier models. Nevertheless, our hypothesis of the episodic-semantic interplay at a neural level requires further experimental study of the synaptic strength dynamics in particular. As mentioned, quantitative data on cortical synaptic plasticity is still quite limited, and while STDP has been shown to offer explanation for some associative memory phenomena (Pokorny et al., 2020), any specific plasticity rule is insufficiently validated experimentally. Yet our results with BCPNN offer a possible explanation and testable behavioral predictions. The spiking version of this plasticity rule has repeatedly been shown to be compatible with detailed, biologically constrained network activity and structure. Importantly, our simulations clearly demonstrate how cognitive phenomena such as semantization could be produced and thus explained by microscopic plasticity processes. In particular, BCPNN solves the issue of decontextualization by its information-theoretical principle, not by being hand-crafted to do so. Like any Bayesian estimator, BCPNN trades-off synaptic strength (weights) for increased intrinsic excitability (bias) in highly active neurons, thus decreasing synaptic strength of neurons that are highly active outside of a specific spiking correlation. Unlike many other conceivable learning rules that might achieve this effect, BCPNN is working only on locally available information, and is thus also biologically plausible.

Our study conforms to related behavioral experiments reporting that high context variability or context overload leads to item-context decoupling (Opitz, 2010; Smith and Manzano, 2010; Smith and Handy, 2014). These studies suggest that context-specific memory traces transform into semantic representations while contextual information is progressively lost. Traces of item memory representations remain intact but fail to retrieve their associated context. Semantization is typically described as a decontextualization process that occurs over time. However, several experiments, including this study, proposed that exposures of stimuli in different additional contexts (rather than time itself) is the key mechanism advancing semantization (Opitz, 2010; Smith and Manzano, 2010; Smith and Handy, 2014). In fact, simple language vocabulary learning implies that learners encode words in several different contexts, which leads to semantization and a definition-like meaning of the studied word (Beheydt, 1987; Bolger et al., 2008). Although our network model is limited to a simple item-context decoupling scenario, the proposed plasticity mechanistic explanation for the observed item-context decoupling effect may be generalized to support semantization in more complex scenarios in which other mechanisms may synergistically interact and contribute to decontextualization. Admittedly, our hypothesis does not exclude other seemingly coexisting phenomena and mechanisms supporting memory retrieval that may facilitate semantization over time, e.g., reconsolidation or systems consolidation because of sleep or aging (Friedrich et al., 2015). Further, our model does not feature any higher-order mechanisms allowing a neutral stimulus (lacking prior pairing) to evoke the same contextual memory response as a conditioned stimulus does despite their prior pairing. In other words, each stimulus has to be independently coupled with its context(s).

**Figure 8.**
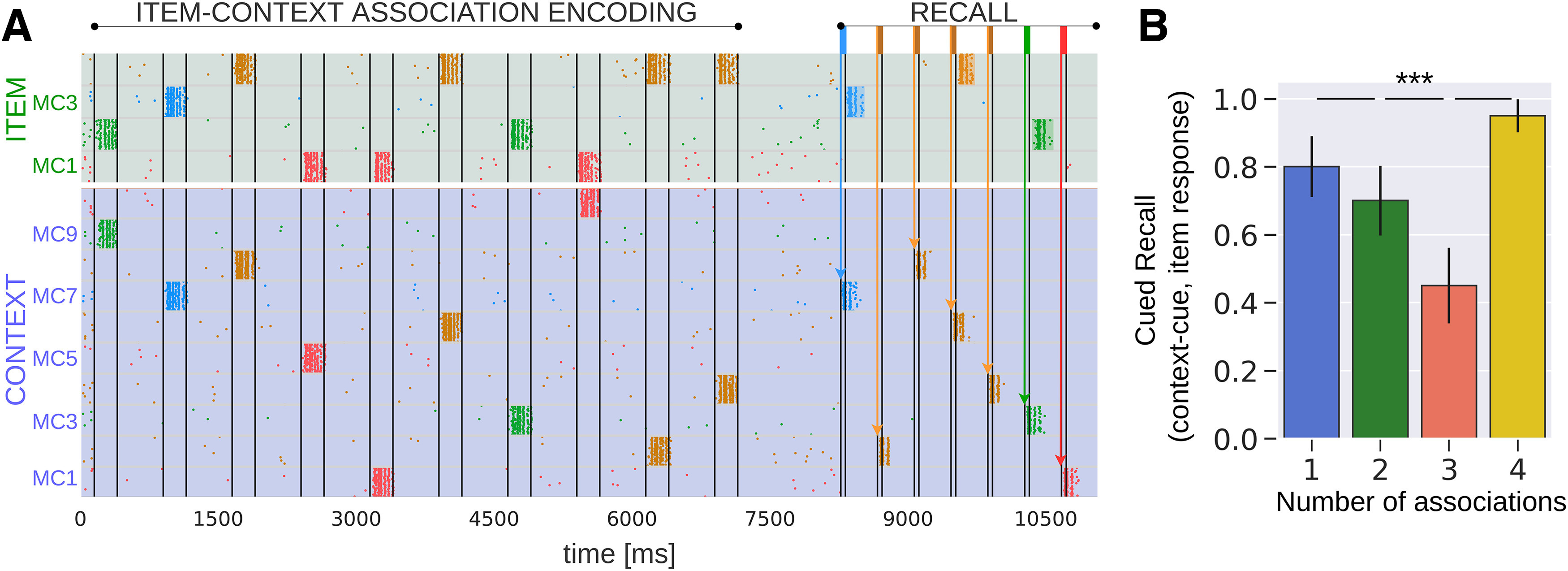
Average cued recall performance in the Item network after sequentially cueing all the contexts that are associated with the item that forms four associations. ***A***, Spike raster of pyramidal neurons in HC1 of both the Item and Context networks. The cue paradigm during test for the one-association, two-association, and three-association case remains identical to the control case (compare Fig. 2*B*). However, in particular for the four-association case, we sequentially cue all the four available contexts that share the same target item. ***B***, Average cued recall performance in the Item network (20 trials). The bar diagram reveals progressive loss of item information over the number of context associations, but not for the four-association case at which all the available contexts were cued during test. Thus, providing more evidence via different sources boosts retrieval (∼95%) recovering a nearly decontextualized item (compare Fig. 3*F*, four associations, single cue, 25% accuracy score); ***p < 0.001 (Mann–Whitney, *N *=* *20). Error bars represent SDs of Bernoulli distributions.

We also demonstrated (Results, Preferential retention) how a transient plasticity modulation, reflecting known isolation effects, may preserve episodicity, staving off decontextualization. Semantization may also be overcome by accumulating additional evidence regarding an episode. In our simulations, we typically used single context cues to retrieve an item during cued recall (e.g., item-4 forms four associations, but only one of its associated contexts was cued, compare Figs. 2*B* and 3*F*). However, an interesting question is whether providing multiple context cues that share the same target item boosts its recall (Fig. 8*A*). Figure 8*B* shows the result where we sequentially stimulated all the four different contexts in the four-association case. The 4-fold contextual information considerably increases the likelihood of retrieval of a nearly fully semanticized item (compare Figs. 3*F* and 8*B*, four associations). These results are relatively intuitive yet novel from a modeling point of view and in line with behavioral studies reporting enhanced cued recall with multiple cues compared with a single one (Rubin and Wallace, 1989; Broadbent et al., 2020; Pearson and Wilbiks, 2021).

To our knowledge, there is no other spiking computational model of comparable detail that captures the semantization of episodic memory explored here, while simultaneously offering a neurobiological explanation of this phenomenon. Unlike other dual-process episodic memory models, which require repeated stimulus exposures to support recognition (Norman and O’Reilly, 2003), our model is able to successfully recall events learned in “one shot” (a distinctive hallmark of episodicity). We note that the attractor-based theory proposed in this study does not exclude the possibility of a dual-process explanation for recollection and familiarity (Yonelinas, 2002; Yonelinas et al., 2010).

### Related models of familiarity and recollection

Perceptual or abstract single-trace dual-process computational models based on signal detection theory explain episodic retrieval but the potential loss of contextual information is only implied as it does not have its own independent representation (Wixted, 2007; Greve et al., 2010). These computational models often aim to explain traditional R/K behavioral studies. As discussed earlier, participants in such studies are instructed to give a “Know” response if the stimulus presented in the test phase is known or familiar without any contextual detail about its previous occurrence. Conversely, “Remember” judgments are to be provided if the stimulus is recognized along with some recollection of specific contextual information pertaining to the study episode. This results in a strict criterion for recollection, as it is possible for a subject to successfully recall an item but fail to retrieve the source information (Ryals et al., 2013). Numerous studies suggest that recollection contaminates “Know” reports because recalling source information sensibly assumes prior item recognition (Wais et al., 2008; Johnson et al., 2009). Mandler (1979, 1980) and Atkinson and Juola (1973) treat familiarity as an activation of preexisting memory representations. Our results are compatible with this notion because our model proposes to treat item-only activations as “Know” judgments, while those accompanied by the activation of context representations best correspond to a “Remember” judgment. Item activation is a faster process and precedes context retrieval (Yonelinas and Jacoby, 1994), and our model reflects this finding by necessity, as item activations are causal to context retrieval.

We assume that familiarity recognition is simply characterized by lack of contextual information, yet the distinction we make between the Context and Item networks is arbitrary. Memory patterns stored in the Context network are referred to as contexts and those in the Item network as items. From the perspective of the network’s architecture, items and contexts have representations of the same nature, nonoverlapping sparse distributed patterns. While sparse internetwork connectivity is sufficient for our model’s function, both networks may just as well be part of the same cortical brain area. The actual physical separation of the two networks (which incurs connection delays commensurate with the axonal conduction speed) is motivated by our assumption that items and contexts are not necessarily represented within the same network. A more specific scenario might assume that items and contexts share part of the same local network. In principle, our model should be capable of replicating similar results in that case.

### Biological plausibility and parameter sensitivity

We investigate and explain behavior and macroscale system dynamics with respect to neural processes, biological parameters of network connectivity, and electrophysiological evidence. Our model consequently builds on a broad range of biological constraints such as intrinsic neuronal parameters, cortical laminar cell densities, plausible delay distributions, and network connectivity. The model reproduces plausible postsynaptic potentials (EPSPs, IPSPs) and abides by estimates of connection densities (i.e., in the associative pathways and projections within each patch), axonal conductance speeds, typically accepted synaptic time constants for the various receptor types (AMPA, NMDA, and GABA), with commonly used neural and synaptic plasticity time constants (i.e., adaptation, depression).

The model synthesizes a number of functionally relevant processes, embedding different components to model composite dynamics, hence, it is beyond this study to perform a detailed sensitivity analysis for every parameter. Instead, we provide insightful observations for previously unexplored parameters that may critically affect semantization. Importantly, a related modular cortical model already investigated sensitivity to important short-term plasticity parameters (Fiebig and Lansner, 2017). After extensive testing we conclude that the model is generally robust to a broad range of parameter changes and its performance only gradually degrades in terms of the effect size. We expect even lower sensitivity to parameter variations in a network approaching biological scales. Further, it is worth reporting that the model’s function is preserved across a wide range of sizes of a cortical column as long as the number of pyramidal cells is not excessively low. The same holds for the population of the inhibitory basket cells provided that the rough total inhibitory synaptic current is maintained by controlling feedback synaptic strength (*g_BP_*; Table 3).

The P trace decay time constant, *τ_p_*, of the BCPNN model is critical for the learning dynamics modelled in this study because it controls the speed of learning in associative connections and the resulting weight amplitude. High values of *τ_p_* imply long-lasting but weaker memory traces and therefore lead to slower, more inertial learning (more resistance to change and encoding new information as well as forgetting) with overall lower weights and hence weaker binding. Varying *τ_p_* by ±30% does not change the main outcome, i.e., episodicity still deteriorates with a higher context variability. At the same time, as mentioned, slower weight development results in weaker associative binding and overall lower recall (and vice versa for faster learning). To compensate for this loss of episodicity, an additional increase in the unspecific input is usually sufficient to trigger comparable recall rates. Alternatively, the recurrent excitatory gain can be amplified to complete noisy inputs toward discrete embedded attractors. Unspecific background input during recall plays a critical role as well. In general, we use a low background noise input into the two coupled networks. However, for the enhanced noise by +40% the model operates in a free recall regime with spontaneously reactivating memories without any external cues.

As we explained in the investigation of the reduced microcircuit model, semantization is an inherent property of BCPNN-driven weight dynamics, derived from Bayesian logic. However, countervailing forces in local microcircuits contribute to the generation and maintenance of our associated memories, Bayesian weight development drives semantization while intrinsic plasticity counteracts it. In consequence, it is possible to lessen the relative impact of this synaptic weight-dependent effect by making intrinsic plasticity more prominent: frequently activated items (in varying contexts) become more excitable because of memory recency effect. Conversely, we can maximize the semantization effect by making the bias current weaker, though fully removing it (*β_gain_* = 0) is hard to justify from a biological perspective. By manipulating the strength of intrinsic plasticity (*β_gain_*) to diminish signs of decoupling as described above, the capacity, i.e., the number of retrievable item-context associations, can increase beyond three associations (compare Fig. 3). Other key factors that can enhance model capacity for item-context associations (resistance to semantization over many established episodic associations) are larger network size, higher associative binding connection probability (e.g., increase in *cp_PPA_* from 2% to 4%; Table 3), and elevated background unspecific noise during the cue-response association period. Strengthening associative binding by upregulating $w_{gain}^{syn}$ can also enhance the model’s capacity for item-context associations (Table 1). Still, there is an upper limit to $w_{gain}^{syn}$ as extreme values can lead to implausible EPSPs.

This study also demonstrates how a selective transient increase of plasticity can counteract semantization. The plasticity of the model can be modulated via the parameter *κ* (Eq. 7; Materials and Methods). Typically, *κ* is set to 1 (*κ* = *κ_normal_*; Table 1), whereas we double plasticity (*κ* = *κ_boost_*; Table 1), when modeling salient episodic encoding. We notice that by selectively tripling or quadrupling plasticity (relative to baseline) during encoding of a specific pair whose item component forms many other associations, the source recall improves progressively (data shown only for *κ* = *κ_boost_* in Results, Preferential retention).

Finally, in Results, BCPNN and STDP learning rule in a microcircuit model, we compare STDP and BCPNN plasticity in a highly reduced model. We bind items with contexts to form different number of associations and keep track of the weight development per time step. STDP plasticity generates same magnitude item-context binding regardless of how many associations an item forms. A detailed parameter analysis for every critical synaptic parameter (±30%) did not yield any behaviorally significant changes to the converged weights.

### Semantization over longer time scales

Source recall is likely supported by multiple independent, parallel, interacting neural structures and processes since various parts of the medial temporal lobes, prefrontal cortex and parts of the parietal cortex all contribute to episodic memory retrieval including information about both where and when an event occurred (Gilboa, 2004; Diana et al., 2007; Watrous et al., 2013). A related classic idea on semantization is the view that it is in fact an emergent outcome of systems consolidation. Sleep-dependent consolidation in particular has been linked to advancing semantization of memories and the extraction of gist information (Payne et al., 2009; Friedrich et al., 2015).

Models of long-term consolidation suggest that retrieval of richly contextualized memories become more generic over time. Without excluding this possibility, we note that this is not always the case, as highly salient memories often retain contextual information (which our model speaks to). Instead, our model argues for a much more immediate neural and synaptic contribution to semantization that does not require slow multiarea systems level processes that have yet to be specified in sufficient detail to be tested in neural simulations. It has previously been shown, however, that an abstract simulation network of networks with broader distributions of learning time constants can consolidate memories across several orders of magnitude in time, using the same Bayesian–Hebbian learning rule as used here (Fiebig and Lansner, 2014). That model included representations for prefrontal cortex, hippocampus, and wider neocortex, implementing an extended complementary learning systems theory (McClelland et al., 1995), which is itself an advancement of systems consolidation (Squire and Alvarez, 1995). We consequently expect that the principled mechanism of semantization explored here can be scaled along the temporal axis to account for lifelong memory, provided that the plasticity involved is itself Bayesian–Hebbian. Our model does not advance any specific anatomic argument as to the location of the respective networks (Yonelinas, 2002; Diana et al., 2007). The model purposefully relies on a generic cortical architecture focused on a class of synaptic plasticity mechanisms which may well serve as a substrate of a wider system across brain areas and time.

In conclusion, we have presented a computational mesoscopic spiking network model to examine the interplay between episodic and semantic memory with the grand objective to explain mechanistically the semantization of episodic traces. Compared with other models of episodic memory, which are typically abstract, our model, built on various biological constraints (i.e., plausible postsynaptic potentials, firing rates, connection densities, synaptic delays, etc.) accounting for neural processes and synaptic mechanisms, emphasizes the role of synaptic plasticity in semantization. Hence it bridges micro and mesoscale mechanisms with macroscale behavior and dynamics. In contrast to standard Hebbian learning, our Bayesian version of Hebbian learning readily reproduced prominent traces of semantization.

## Synthesis

Reviewing Editor: Niraj Desai, National Institute of Neurological Disorders and Stroke Decisions are customarily a result of the Reviewing Editor and the peer reviewers coming together and discussing their recommendations until a consensus is reached. When revisions are invited, a fact-based synthesis statement explaining their decision and outlining what is needed to prepare a revision will be listed below. The following reviewer(s) agreed to reveal their identity: Aditya Singh.

The manuscript has now been read by two expert reviewers. Both find the study contains interesting and important insights, but both also have a number of detailed questions, comments, and suggestions for improvement. Please address each point listed below in your resubmission.

REVIEWER #1

In this study, authors built a cortical spiking neural network model to simulate the semantization phenomenon. They found that the traditional STDP learning rule could not explain the decontextualization process while BCPNN could. The study is interesting and presented several new insights on the episodic and semantic memory processes.

However, I have some concerns as listed below:

1. In Table1, authors listed several parameter values used in the study. Since this is a model study, can author add information (e.g. figures) about what biological observations does the model(s) match. For example, do the adaptation-related parameters help single cell model match the F-I curve of single cell? In other words, before going to the network, how did author calibrate the single cell model/synapse?

2. What is the rationale for choosing the number of neurons to model, i.e., line 162, authors use 30 pyramidal cells to model each minicolumn.

3. In table 3, does the connectivity value mean the mean connectivity from a randomized connectivity distribution in the network?

4. Section “Biological plausibility and parameter sensitivity” can be moved into Discussion section.

5. Author mentioned several times about the oscillatory part of the model. But I couldn’t find where it is presented in the results session.

6. Even though authors did not omit the discussion of parameter sensitivity study, sweeping some important parameters should be critical for showing in-depth understanding of why BCPNN can favor in the model having semantization phenomenon, and more importantly, what key features does the BCPNN have that could regulate this phenomenon. For example, can author show that by changing the value of parameters of BCPNN, the progressive loss of episodic context information can become more/less radical? And these kind of parameters might provide useful biology-related insights. See some example publications (might not from the same field, but idea is certain phenomena can be regulated by changing some parameters.): Rao-Ruiz et al., 2019 (Current Opinion in Neurobiology), Feng et al., 2016 (Neuroscience) and Morrison et al., 2016 (Neurobiol Learning Mem.)

REVIEWER #2

In this study titled, “Traces of semantization - from episodic to semantic memory in a spiking cortical network model", authors use a spiking neural network model with Bayesian Confidence Propagation Neural Network (BCPNN) to investigate the role of Bayesian-hebbian synaptic plasticity in semantization or item-context decoupling after simulated learning. Building on previous knowledge in this field, especially when the field has limited data on cortical synaptic plasticity, authors take an important step forward to improve our understanding of the synaptic basis of mnemonic processes in cortical networks. The study offers interesting insights on learning, specifically for the case when an item is encoded in multiple contexts leading to memory decontextualization. The findings presented in the study speak to a broad audience and I believe such results can motivate further quantitative investigations of synaptic basis of semantization of memories. I enjoyed reading the manuscript as it is engaging, clearly written, and the information flows well from the beginning to the end, however, I also think there are some aspects in the study that need some revision to improve the clarity and make the results more accessible to the readers.

Note: I am listing my concerns in the order as they appear in the manuscript from beginning to end.

Line# 111: The meaning of the word “trace” is not clear at this point and it can be explained/defined here briefly.

Table 1 and 2: The number of parameters for BCPNN is 28, while for the STDP model it is only 8. So for a fair comparison in the reader’s mind, authors should also bring out the aspect that STDP provides parsimonious explanations for several mnemonic phenomena.

Line 160-162: What is the basis for stating that one active MC per HC approximates the sparse neocortical activity with marginal overlap? Appropriate citations may be added here.

Line 163-165: Is there a reference/explanation for choosing the stated number of excitatory and inhibitory neurons in the simulated network? Does this E/I ratio (7200/480) match the in vivo observations in cortical networks (references?)?

Line 179-181: How is the effect of not-explicitly simulated double-bouquet cells expressed by BCPNN rule? The expressed effect is equivalent to approximately how many double bouquet cells in an MC/HC?

Line 181-183: What does “similar model architecture” mean and how do authors estimate the similarity of their model with the model used in Chrysanthidis et al 2019?

Table 3:

"Basket cells per MC - value 2”: Does this number match the in vivo E/I ratio? on what basis was this value chosen?

"Distance between networks - value 10mm”: How does changing this distance affect the model performance, for example, to match the distance between visual cortical areas in the human brain? Since authors simulate a primarily visual behavior task from Opitz et al 2010, does this distance match the network size of visual processing? This would have implications for extending our understanding gained from this study to other behavior tasks incorporating multiple modalities.

Figure 1A: How are preloaded memories of item and context representations defined as long term? Are the weights for such long-term memory networks resistant to change during the encoding of new information? Are these the same as the item and context attractors embedded in each network that remain fixed during simulation representing long-term well-consolidated memories?

Figure 1C: What do the authors mean by the statement that, “inhibition is overall balanced between patterns (blue)”? What is the meaning of “pattern” here?

Line 194: How did authors arrive at the number 1.5ms for the minimal delay to reflect synaptic delays due to effects that are not explicitly modeled?

Line 232: What do authors mean by “model degrades gracefully”?

Lines 236-237 and 238-239: These two lines seem to be making contradictory statements as the first line states that slower learning is long-lasting while the second states that slower weight development may result in weaker associative binding. It would help to make this clearer for the reader.

Line 243: The phrase “ Low such noise” could be written more clearly.

Line 253: What are the parameters in the highly reduced model?

Line 254-255: What about BCPNN plasticity in the highly reduced model? Was it the same as that for STDP?

Figure 2: How are different contexts simulated as “different context” in terms of network activation? By stimulating completely different HC-MC networks or by different yet somewhat overlapping patterns of ensembles within HC/MCs, or a different pattern of weight distributions?

Can the degree of similarity across all the simulated contexts be quantified? For example, by disintegrating each context into its component features and then estimating the number of common features among contexts to classify them on a scale of increasing similarity?

When the same item is experienced in multiple contexts, there are higher-order mechanisms (refer to higher-order conditioning literature) that can strengthen the associations. Authors should try to incorporate such higher-order conditioning mechanisms in the model or discuss the limitation of this model in incorporating higher-order learning processes.

What is the significance of the “1-second” delay between encoding and recall? How could a different (longer/shorter) delay affect the recall?

Figure 2B: Is context expected to incorporate a larger network than the item? Is it possible to scale the duration of exposure to context during retrieval according to the ratio of stimulated network sizes for context vs item? For example, if an item requires stimulation of a network with ‘i’ neurons and context requires ‘c’ neurons, should the duration of exposure to context during retrieval be scaled by the ratio ‘c/i’? Considering that context recall requires larger network activation than item recall, authors should comment on the effect of duration of exposure to item- vs context-cues during recall.

Line 284-285 (and Line 325-327): Current model is limited to the successful cued recall of only up to three item-context associations. Although it is not clear which parameters in the model are the key contributor to this limit. To improve the understanding of the model, it would help if authors can assess and provide more information about the key parameters (other than the size of the network) that can be changed in the model to increase the number of retrievable item-context associations from 3 to, say, 4,5, or 6?

Line 289-290: The statement about the failure of context recall could be rephrased as it seems repetitive.

Line 300-301: Item-context decoupling may only be one aspect of episodic memory as items memorized in a sequence within the same context can also form an episodic memory (for example Howard Eichenbaum lab’s odor-sequence task). So authors should be careful in replacing the observed item-context decoupling with terms such as semantization or loss of episodicity.

The definition of semantization is not clear in the field so far. Also, it is not known whether semantization is a biological limitation in retrieval or encoding. None of the previous studies, including the ones cited in the manuscript, have given conclusive evidence that loss of episodicity is associated with the weakening of associative synaptic weight distributions. So authors should clarify this limitation that the field of learning and memory is facing and the observed effects in this modeling study may not be the basis of behaviorally observed semantization. Authors do make a statement regarding this in discussion (line 432-433, I suppose) although it would be insightful for the reader to mention such limitation in this results section wherever conclusions are being drawn from data.

Fig 3A: Why do item and context attractors (solid red lines) do not stop/oppose item-context decoupling?

Authors should discuss whether previous literature suggests decoupling arises due to inefficient retrieval of memories or inefficient encoding of memories?

Fig 3B: During behavior, every item is always experienced in some context. So the model should incorporate partial context-cues along with items to closely resemble behavior. What would the model output be if each item is cued along with partial context cue, even for 4-association case?

Fig 3C and 3F: Which factors lead to relatively higher recall for 4-associations when context is cued (3F) as compared to when item is cued (3C)?

Fig 4B-4D: Why is there a difference in cued recall for one association case between item-cue (4B) and context-cue (4D)? Why this difference is not observed in BCPNN model?

Fig 6: Is the final readout spike at 11sec a spontaneous spike? Why does it occur at 11sec?

Line 399: What happens when kappa is increased to kappa-boost for one item-four context scenario?

Fig 7A: Why do the 4-association (yellow) and 2-association (orange) bars change from normal to biased conditions? Why 1- (blue) and 2-association (green) bars don’t change from normal to biased scenarios?

Fig 8: How does recall change from 4-context cued recall to 3- or 2-context cued recall?

Line 493: It’s not clear what authors mean by any item can be a context. Do they mean any item network can encode context or something else?

Line 497 - Until this line in the manuscript, it was not clear if the authors were simulating a single modality scenario or a multiple modality scenario. In fact, based on the value (10mm) of parameter “Distance between networks” in table 3, the reader may preemptively assume that authors were simulating a single modality scenario.

Line 506: It may make the sentence more clear if it is modified to, “ Models of long-term consolidation suggest that retrieval of richly contextualized memories...”

## Author Response

Dear eNeuro Editor,

We hereby submit a revised version of our manuscript (eN-NWR-0062-22). We have considered the Reviewers’ comments and Editor’s instructions and applied modifications and additions to the text accordingly.

Below we provide our replies to Reviewer’s comments as they listed. We hope these clarifications and additions make our manuscript acceptable for publication in eNeuro.

We would further like to clarify that the reported line numbers in this letter correspond to the line numbers of the annotated Article file provided in Word format which includes insertions (marked as underlined text) and deletions (marked as crossed out text).

Best regards,

The corresponding author of the manuscript

______________________________________________________________________________

REVIEWER #1

In this study, authors built a cortical spiking neural network model to simulate the semantization phenomenon. They found that the traditional STDP learning rule could not explain the decontextualization process while BCPNN could. The study is interesting and presented several new insights on the episodic and semantic memory processes.

However, I have some concerns as listed below:

1. In Table1, authors listed several parameter values used in the study. Since this is a model study, can author add information (e.g. figures) about what biological observations does the model(s) match. For example, do the adaptation-related parameters help single cell model match the F-I curve of single cell? In other words, before going to the network, how did author calibrate the single cell model/synapse?

Reply: Our model is a composite of many well established models. In fact most of the parameters in this table are commonly accepted default parameters in the field that we take as a given. For example, we use the adaptive exponential integrate-and-fire model developed by Brette and Gerstner (2005) as a very effective model of cortical neuronal activity, reproducing a wide variety of electrophysiological properties. Because this is such a well established and highly influential model with carefully validated parameters we did not attempt any specific validation ourselves and used default values for pyramidal neuron’s parameters as a given (with minor exceptions, which we point out, such as adding an additional current to implement intrinsic plasticity or removing the subthreshold adaptation of that model). Similarly, many of the other parameters are simply standard values from their underlying publications or commonly accepted computational fits for cortical pyramidal cells. For example, short-term synaptic plasticity parameters pertaining to depression and facilitation are drawn from the range of proposed parameters of the underlying Tsodyks-Markram model (Tsodyks et al., 1997) and well-designed fits of that model to electrophysiological recordings of cortical pyramidal cells (Wang et al., 2006). In addition, we performed sensitivity analysis for a selected subset of parameters and the results are described in section “Biological plausibility and parameter sensitivity”. However, in response to the Reviewer’s question we made sure to highlight these underlying component models and their parameters explicitly throughout the manuscript (e.g. lines 77-83, 98-100) and also separated the different parameters in Table 1 more clearly into 3 discrete column/categories to avoid the impression that our model has a plethora of novel or ill-constrained model parameters. Please also see also our responses to Reviewer#2 on the topic of simulation parameters (comment no. 2).

2. What is the rationale for choosing the number of neurons to model, i.e., line 162, authors use 30 pyramidal cells to model each minicolumn.

Reply: Each functional cortical minicolumn in primates contains ∼80-100 neurons (Peters and Yilmaz, 1993; Mountcastle, 1997). The layer 2/3 that we simulate essentially occupies the outer one-third of the thickness of the cortex, and thus we represent it by simulating 30 pyramidal cells for each cortical column. We have clarified this in the manuscript now (lines 177-179). We would also like to note that the model’s function is preserved across a wide range of the sizes of a cortical column as long as the number of neurons is not excessively low (Biological plausibility section: lines 515-517).

3. In table 3, does the connectivity value mean the mean connectivity from a randomized connectivity distribution in the network?

Reply: Connectivity values in Table 3 refer to the fixed (it is not a parameter of the random distribution) proportion of connections that are drawn between two populations (source and target layer). Each neuron in the target layer is visited and sources for it are selected from the source layer based on a fixed probability. In other words, connectivity value denotes the chance that randomly drawn connection pairs exist (see change in lines 185-190).

4. Section “Biological plausibility and parameter sensitivity” can be moved into Discussion section.

Reply: Thank you for this suggestion, we have considered this before. We are happy to move it into Discussion, as suggested.

5. Author mentioned several times about the oscillatory part of the model. But I couldn’t find where it is presented in the results session.

Reply: Thank you for the comment - we recognise now the risk for confusion. Indeed, we do not specifically show this aspect of the model in the results section. Consequently, given that it is not related to any particular functional aspect of relevance to the purpose of our model study, we simply decided to delete any mention of oscillations (e.g., in lines 231-232, 412-413, 504-506).

6. Even though authors did not omit the discussion of parameter sensitivity study, sweeping some important parameters should be critical for showing in-depth understanding of why BCPNN can favor in the model having semantization phenomenon, and more importantly, what key features does the BCPNN have that could regulate this phenomenon. For example, can author show that by changing the value of parameters of BCPNN, the progressive loss of episodic context information can become more/less radical? And these kind of parameters might provide useful biology-related insights. See some example publications (might not from the same field, but idea is certain phenomena can be regulated by changing some parameters.): Rao-Ruiz et al., 2019 (Current Opinion in Neurobiology), Feng et al., 2016 (Neuroscience) and Morrison et al., 2016 (Neurobiol Learning Mem.)

Reply: Our sensitivity analysis accounts for key BCPNN parameters in the study. With regard to the semantization effect itself, it is an inherent property of BCPNN-driven weight dynamics resulting from the Bayesian logic, as explained also in the investigation of the reduced microcircuit model (Section: BCPNN and STDP learning rule in a microcircuit model). The effect size can be less radical by making the intrinsic plasticity component of the learning rule more prominent, that is, frequently activated items (activated in variable context) become more excitable due to the memory recency effect. Conversely, we can maximize the semantization effect by making the bias current (intrinsic plasticity) weaker. In response to the Reviewer’s comment, we now added this aspect to our discussion under the heading of biological plausibility and parameter sensitivity (lines 535-542).

______________________________________________________________________________

REVIEWER #2

In this study titled, “Traces of semantization - from episodic to semantic memory in a spiking cortical network model", authors use a spiking neural network model with Bayesian Confidence

Propagation Neural Network (BCPNN) to investigate the role of Bayesian-hebbian synaptic plasticity in semantization or item-context decoupling after simulated learning. Building on previous knowledge in this field, especially when the field has limited data on cortical synaptic plasticity, authors take an important step forward to improve our understanding of the synaptic basis of mnemonic processes in cortical networks. The study offers interesting insights on learning, specifically for the case when an item is encoded in multiple contexts leading to memory decontextualization. The findings presented in the study speak to a broad audience and I believe such results can motivate further quantitative investigations of synaptic basis of semantization of memories. I enjoyed reading the manuscript as it is engaging, clearly written, and the information flows well from the beginning to the end, however, I also think there are some aspects in the study that need some revision to improve the clarity and make the results more accessible to the readers.

Note: I am listing my concerns in the order as they appear in the manuscript from beginning to end.

1. Line# 111: The meaning of the word “trace” is not clear at this point and it can be explained/defined here briefly.

Reply: First and foremost, synaptic traces meant here should not be confused with an abstract term “traces” in the title. Synaptic traces, noted as Z, and P in the manuscript, refer to cascades of synaptic processes at varying time scales, τz , and τp , respectively, caused by pre- and post-synaptic spikes. They can be thought of as “memory” traces of spiking events (reflecting their temporal integration), which have direct impact on changes of synaptic weights and biases through the BCPNN formalism. From a theoretical perspective, BCPNN interprets P traces as a temporal estimate of probabilities of neuron activations (as well as pair-wise co-activations), derived as the exponential average of Z traces. The effect of these dynamical variables, Z, and P, on BCPNN weights and biases is described in the manuscript. In response to the Reviewer’s question, we have added a clarification that makes the meaning of Z, and P traces more intuitive. In particular, the sentence in section Spike-based BCPNN plasticity reads now as follows (lines 114-117): “Specifically, learning implements exponential filters, Z, and P, of spiking activity with a hierarchy of time constants, τZ, and τP, respectively (the full BCPNN model implements additional eligibility E traces [Tully et al. (2014)], which are not used here). Due to their temporal integrative nature they are referred to as synaptic (local memory) traces”. 2. Table 1 and 2: The number of parameters for BCPNN is 28, while for the STDP model it is only 8. So for a fair comparison in the reader’s mind, authors should also bring out the aspect that STDP provides parsimonious explanations for several mnemonic phenomena.

Reply: Thank you for this comment. We understand the Reviewer’s concern. At the same time, we would like to clarify that there are only 8 BCPNN parameters (fmin , fmax , epsilon, τz , τP, K, wgain , βgain ). We understand how this impression of a plethora of BCPNN parameters came about because in Table 1 we lumped together the BCPNN model parameters with the parameters of the AdEx neuron model by Brette & Gerstner, and the STP mechanism by Tsodyks & Markram, etc. Most of these parameters are obviously also used in the STDP-based model, which merely replaces the BCPNN learning rule. We made some adjustments to both the parameter table and the manuscript text to clarify this (see earlier answer to Reviewer#1 [comment no. 1]). We recognize that STDP is useful for explaining some associative memory phenomena (see added line 416-417) “STDP has been shown to offer explanation for some associative memory phenomena (e.g. Pokorny et al., 2020)”.

This comparative aspect is indeed of crucial importance. As the Reviewer rightly pointed out, the standard STDP rule used here is defined by a lower number of parameters than our BCPNN learning rule. However, the difference is not that striking as one might think based on Table 1. To avoid this misinterpretation we have now restructured Table 1 into "Neuron model parameters", Receptor parameters", “BCPNN parameters", and “Short-term plasticity parameters”. As can be seen, the number of BCPNN parameters per receptor type (AMDA, NMDA) is 8.

3. Line 160-162: What is the basis for stating that one active MC per HC approximates the sparse neocortical activity with marginal overlap? Appropriate citations may be added here.

Reply: In our model the proportion of neurons encoding a memory pattern within each hypercolumn defines the level of sparseness. Given the model assumption that only a single minicolumn per hypercolumn can be activated, the sparseness level reflects the reciprocal of the number of minicolumns per hypercolumn. Thanks to the Reviewer’s comment we recognise that the given sentence in the manuscript may be confusing so we slightly edited this part in the manuscript to be more clear, and provide some references (lines 172-177): “Our item and context memory representations are distributed and non-overlapping, i.e. with a single distinct pattern-specific (encoding) MC per HC. This results in sparse neocortical activity patterns (Barth & Poulet 2012). It should be noted that the model tolerates only a marginal overlap between different memory patterns, i.e. shared encoding minicolumns (data not shown).” Neocortical activity is sparse (Barth & Poulet 2012). Recurrently connected neurons in layer 2/3 of a functional column often share the same selectivity and synaptic inputs (Yoshimura et al. 2005). Our model reflects this fine-scale structure by having item/context selective minicolumns. In the biological neocortex there are ∼80 functional columns in each cortical macrocolumn (Mountcastle, 1997), so we assume that a full-scale model would naturally approach very sparse activity with minimal overlap between selective cell assemblies.

4. Line 163-165: Is there a reference/explanation for choosing the stated number of excitatory and inhibitory neurons in the simulated network? Does this E/I ratio (7200/480) match the in vivo observations in cortical networks (references?)?

Reply: Our model represents explicitly only one type of inhibitory interneurons (i.e. basket cells). So at first glance our model does not match in-vivo observations of a 4:1 or 5:1 ratio of excitatory to inhibitory cells often assumed in simpler, random unstructured networks.

However, as we simulate the extent of layer 2/3, and explain in lines 177-178 (and in caption of Fig. 1), and illustrate in Figure 1, our model also accounts for another type of inhibition - namely, disynaptic inhibition mediated via dendritic targeting double bouquet and/or bipolar cells. As a result, a sizable fraction of the total inhibition (i.e. all the “learned" inhibition) is modeled implicitly via learned weights rather than explicitly via inhibitory cells.

Network models with negative synaptic weights have been shown to be functionally equivalent to ones with both excitatory and inhibitory neurons with only positive weights (Parisien et al., 2008). Following this approach, it was specifically validated in the previous work (Chrysanthidis et al. 2019) that learned mono-synaptic inhibition between competing attractors is functionally equivalent to the disynaptic inhibition mediated by double bouquet and basket cells.

In fact, the model performance is not sensitive to the number of interneurons, provided that the rough total of the inhibitory synaptic current is maintained. To clarify this possible point of misunderstanding, we rephrased and extended the corresponding paragraph, see lines 197-202, 517-519. 5. Table 3: “Basket cells per MC - value 2”: Does this number match the in vivo E/I ratio? on what basis was this value chosen?

Reply: Thank you for the question. In our view, the E/I ratio is a coarse metric lumping together all excitatory and inhibitory cells into their respective group without distinguishing functionally different forms of inhibition (please see also our response to the preceding comment no. 4, which explains why our overall I-cell counts are low). We have chosen the number of basket cells on the basis of its commonly assumed function. In neocortex, about half of all inhibitory interneurons are basket cells, specializing in targeting the somata and proximal dendrites of pyramidal neurons. This places them in a unique position to adjust the gain of the integrated synaptic response (Markram, 2004). Their broad, yet dense, reciprocal connectivity with pyramidal cells within their entire hypercolumn and their high IPSP amplitude (Thomson et al., 2002) make them provide the lion’s share of fast (lateral) feedback inhibition. Therefore, these cells are ideally placed to implement a kind of winner-take-all mechanism in the cortical column through competition of constituent functional (mini)columns. We have decided on scaling the number of basket cells by the number of simulated minicolumns to maintain this functional role even if we scale the number of simulated functional columns per hypercolumn. The amount of feedback inhibition mostly depends on the pyramidal-basket-pyramidal connection probabilities (cpPB and cpBP), and the inhibitory conductances of each basket cell connection (gBP), which are well constrained by electrophysiological data (Thomson et al., 2002). We have also determined that 2 basket cells per MC are sufficient for them to fulfill their assigned role within the hypercolumn at these values. However, the model is not particularly sensitive to the exact number of basket cells. We explicitly tested this by doubling the basket cell count and we did not observe any particular change in activity patterns. So, as long as the overall amount of feedback inhibition within the hypercolumn stays above a minimal threshold required to implement its fast feedback function, the model operates in the same regime.

As pointed out in our response to question no. 4, we have clarified this in the Discussion of the revised manuscript.

6. Line 179-181: How is the effect of not-explicitly simulated double-bouquet cells expressed by BCPNN rule? The expressed effect is equivalent to approximately how many double bouquet cells in an MC/HC?

Reply: BCPNN describes the effect of not-explicitly simulated double-bouquet cells (DBCs) by replacing disynaptic inhibition with negative connections (GABA reversal potential) between cell assemblies that do not share the same pattern selectivity (see response to the two previous comments no. 4, 5) There is almost exactly one DBC per MC in monkey and human cerebral neocortex (Javier DeFelipe et al. 2006), innervating its entire functional (mini)column with thin vertical “horsetail-shaped axons", which is in line with our assumption that DBCs mediate learned inhibition for their entire functional column.

7. Line 181-183: What does “similar model architecture” mean and how do authors estimate the similarity of their model with the model used in Chrysanthidis et al 2019?

Reply: Thank you for pointing this out - we concur that the formulation is not specific enough. While the model by Chrysanthidis et al. (2019) is not a model composed of two interlinked networks, each component network uses the same conductance-based AdEx neuron model, follows the same cortical architecture with modular structure and pattern selective MCs, and features the same cell counts per column, resulting in nearly identical microcircuits.

In response to the Reviewer’s comment we have now more precisely referred to the previous work in the manuscript, please see lines 198-202: “A recent study based on a similar single-network architecture (i.e. with the same modular organisation, microcircuitry, conductance-based AdEx neuron model, cell count per MC and HC) demonstrated that learned mono-synaptic inhibition between competing attractors is functionally equivalent to the disynaptic inhibition mediated by double bouquet and basket cells (Chrysanthidis et al., 2019).” 8. “Distance between networks - value 10mm”: How does changing this distance affect the model performance, for example, to match the distance between visual cortical areas in the human brain? Since authors simulate a primarily visual behavior task from Opitz et al 2010, does this distance match the network size of visual processing? This would have implications for extending our understanding gained from this study to other behavior tasks incorporating multiple modalities.

Reply: Longer delays (and possibly lower connection probabilities) would reduce the overall retrieval score over the number of associations. However, since the integration time for activations and attractor dwell times are rather long (∼100ms) when compared to connection delays, the qualitative model outcomes and semantization effect in particular would not be affected (unless the connection delays are at least one order of magnitude longer). With regard to the simulated memory modalities, please see our answer to the next question (no. 9).

9. Line 497 - Until this line in the manuscript, it was not clear if the authors were simulating a single modality scenario or a multiple modality scenario. In fact, based on the value (10mm) of parameter "Distance between networks” in table 3, the reader may preemptively assume that authors were simulating a single modality scenario.

Reply: We agree with the Reviewer that we have not been sufficiently clear in how we refer to the two simulated networks in the context of the brain’s organization. The way we originally phrased it is indeed misleading. It is a matter of interpretation whether the two networks simulated are indeed located in separate modalities or different areas of the visual hierarchy. Unlike some related studies (see Fiebig et al. 2020), we do not explicitly simulate hierarchy, or hierarchical projections, nor do we make any specific anatomical claims about which cortical patches might be represented by the two simulated networks. The choice of two connected networks was motivated by the general task design. Items and tasks/contexts are visually presented to human subjects in Opitz (2010) study. So, in this particular paradigm we can think of the two networks as both residing somewhere in the visual hierarchy. However, they might as well belong to different modalities. The distance of 10mm is somewhat arbitrary and not particularly constrained. The actual physical separation of the two networks (which incurs connection delays commensurate with the axonal conduction speed) is motivated by our assumption that items and context memories are not necessarily represented within the same network. As mentioned in our answer to the previous question (no. 8), extending connection delays would not have a qualitative effect on the model outcomes or semantization findings.

In response to the Reviewer’s observation we have extended and rephrased the paragraph in question to avoid any impression that we simulate specific areas or modalities (see lines: 484-495). 10. Figure 1A: How are preloaded memories of item and context representations defined as long term? Are the weights for such long-term memory networks resistant to change during the encoding of new information? Are these the same as the item and context attractors embedded in each network that remain fixed during simulation representing long-term well-consolidated memories?

Reply: Memory items and contexts were encoded off-line and preloaded (Fig. 1), i.e. prior to the simulations reported in the Results. The encoding was performed with slow BCPNN learning (very long τP) and the resulting intra-network pattern encoding synaptic weights were fixed during the subsequent main simulations of the two-network model (except for non-Hebbian fast depression and augmentation effects). These intra-network weights represent long-term well-consolidated item and context memories in their respective networks. Consequently, they were resistant to changes during associative learning (of inter-network weights) between Item and Context networks. In response to the Reviewer’s comment, we clarified this further in lines 167-172.

11. Figure 1C: What do the authors mean by the statement that, “inhibition is overall balanced between patterns (blue)”? What is the meaning of “pattern” here?

Reply: Patterns refer to encoded item memories in the Item network and context memories in the Context network. The Item and Context network architecture follows a modular cortical structure with the winner-take-all type of competition within each hypercolumn (HC). This competition is partly mediated by disynaptic inhibition (Fig. 1B), which is distributed equally across the MCs within and between HCs, and thereby across different cell assemblies representing memory (item/context) patterns. In response to the Reviewer’s comment we acknowledge that this phrase “inhibition is overall balanced between patterns (blue)” can raise confusion so we decided to delete the particular sentence from the Figure 1C caption.

12. Line 194: How did authors arrive at the number 1.5ms for the minimal delay to reflect synaptic delays due to effects that are not explicitly modeled?

Reply: Thomson et al. (2002, Table 1) reported an average latency of 1.5 ms for neighboring pyramidal cells in layer 2/3. There are many biophysical mechanisms, e.g. synaptic and dendritic passive conduction, that affect this delay. We take this to mean that there is a distance-independent offset of 1.5 ms in the otherwise distance-dependent connection latency.

13. Line 232: What do authors mean by “model degrades gracefully”?

Reply: Thank you for this comment - this is a jargon term used in computational science that should be avoided here. We simply mean that the model’s performance/function gradually degrades rather than suddenly collapsing. In the manuscript we particularly referred to the fact that extending the synaptic plasticity time constant (τp ) resulted in gradual decay in performance due to slower learning and thus overall weaker binding. Yet, the item-context decoupling could be observed over the higher number of associations.

Consequently, in response to the Reviewer’s comment we have changed the formulation in the manuscript (lines: 511-513): “After extensive testing we conclude that the model is generally robust to a broad range of parameter changes and its performance only gradually degrades in terms of the effect size”.

14. Lines 236-237 and 238-239: These two lines seem to be making contradictory statements as the first line states that slower learning is long-lasting while the second states that slower weight development may result in weaker associative binding. It would help to make this clearer for the reader.

Reply: In BCPNN, slower learning implies long-lasting memory traces due to longer τp . In essence, the learning has inertia and weights are resistant to encoding new information (and hence forgetting). It can be interpreted as learning with lower learning rate. In consequence, the weights subject to slow learning, i.e. with long τP, are weaker since longer-lasting memory traces have lower amplitude. Weights could be made stronger by repeated training of the given associations (corresponding to multiple epochs).

In response to the Reviewer’s comment we have further clarified this in lines 520-524.

15. Line 243: The phrase “ Low such noise” could be written more clearly.

Reply: We use a low background noise input to the two networks to model cue-association responses. We agree with the suggestion and in response to the Reviewer’s point we reshaped this sentence accordingly in lines (530-534) “In general, we use a low background noise input into the two coupled networks. However, for the enhanced noise by +40% the model operates in a free recall regime with spontaneously reactivating memories without any external cues.” 16. Line 253: What are the parameters in the highly reduced model?

Reply: The neural and synaptic parameters (and most importantly all the plasticity parameters) used for the highly reduced model are identical to the ones used for the large scale model (see Table 1, 3). The difference is just in the scale/architecture (just one network, no columns, just one selective cell per item/context, and longer stimulation times to highlight weight development dynamics). We have added parts of this reply in the manuscript to better elucidate this question, lines 341-343, “The neural and synaptic parameters (and most importantly all the plasticity parameters) used for the highly reduced BCPNN and STDP model are identical to the ones used for the large scale BCPNN and STDP model, respectively (see Table 1, 2)”. 17. Line 254-255: What about BCPNN plasticity in the highly reduced model? Was it the same as that for STDP?

Reply: The parameters used for the highly reduced model are identical to the ones used for the larger scaled simulated model (see Table 1, 2). This applies to both BCPNN and STDP network models. Please, see also our reply to the preceding comment no. 16. 18. Figure 2: How are different contexts simulated as “different context” in terms of network activation? By stimulating completely different HC-MC networks or by different yet somewhat overlapping patterns of ensembles within HC/MCs, or a different pattern of weight distributions?

Reply: Our memory patterns in the Context network as well as those in the Item network have no overlaps in their network representations. Each context/item pattern corresponds to the activation of a unique set of minicolumns in its network. Our stimulation is received by neurons within these pattern-specific distinct minicolumns so different contexts are stimulated by their distinct cues. We have now clarified this point in Figure 3B caption when explaining the spike raster of pyramidal neurons in HC1 of both the Item and Context networks. Our response to the Reviewer’s comment no. 3 is also relevant here. 19. Can the degree of similarity across all the simulated contexts be quantified? For example, by disintegrating each context into its component features and then estimating the number of common features among contexts to classify them on a scale of increasing similarity?

Reply: As mentioned above (responses to the Reviewer’s comments no. 3 and 18), all memory representations including context patterns are non-overlapping so there is no similarity in terms of shared features. In fact, the representation aspect of our model is abstract and does not rely on any feature representations. 20. When the same item is experienced in multiple contexts, there are higher-order mechanisms (refer to higher-order conditioning literature) that can strengthen the associations. Authors should try to incorporate such higher-order conditioning mechanisms in the model or discuss the limitation of this model in incorporating higher-order learning processes.

Reply: Thank you for raising this interesting point. However, we have not seen any connection to higher order learning processes in the experimental literature on episodic memory and semantization. Still, we are convinced that such relations exist in reality and admittedly our model in the current form does not account for them. If we wanted to represent such phenomena in this limited model we would need to add complexity that would hide the main message of our contribution. In principle, our model could reproduce such high-order mechanisms, yet the task set up is a R/K experiment without including conditioned stimuli (Opitz, 2010). In line with the Reviewer’s suggestion we have added text in lines 445-447 in the Discussion section to highlight such limitations of the current model. "Further, our model does not feature any higher-order mechanisms allowing a neutral stimulus (lacking prior pairing) to evoke the same contextual memory response as a conditioned stimulus does. In other words, each stimulus has to be independently coupled with its context(s).” 21. What is the significance of the “1-second” delay between encoding and recall? How could a different (longer/shorter) delay affect the recall?

Reply: The model does not rely on short-term plasticity dynamics for information storage, but it rather builds on BCPNN synaptic associative traces at longer time constants. Therefore, only after longer delays we expect the model performance to degrade. At the same time, in biological reality there could be several processes i.e., short-term plasticity mechanisms, noise, unintended distractors etc., which could degrade performance during the delay period. 22. Figure 2B: Is context expected to incorporate a larger network than the item? Is it possible to scale the duration of exposure to context during retrieval according to the ratio of stimulated network sizes for context vs item? For example, if an item requires stimulation of a network with ‘i’ neurons and context requires ‘c’ neurons, should the duration of exposure to context during retrieval be scaled by the ratio ‘c/i’? Considering that context recall requires larger network activation than item recall, authors should comment on the effect of duration of exposure to item vs context-cues during recall.

Reply: Thank you for this interesting thought. Contexts may indeed recruit a larger network or networks in the cortex. On the other hand, it does not always have to be the case, as context may also be considered as just any additional episodic detail related to an item. In Opitz’ (2010) study a context is defined as the location of the given item on the screen or the word that describes the task that an item participates in (e.g., indoor item). All in all, we agree with the Reviewer that contexts and items are in general different and can be experienced on different time scales. Taking this aspect into account however remains outside the scope of the proposed model. When it comes to the sheer matter of timing of the stimulus in our network model, it is important to point out that synaptic integration across the stimulated cell assembly (context or item) happens on the order of tens of ms, not on behavioral time scales. In fact, the assembly recruitment and pattern activation hardly depends on the size of our network. 23. Line 284-285 (and Line 325-327): Current model is limited to the successful cued recall of only up to three item-context associations. Although it is not clear which parameters in the model are the key contributor to this limit. To improve the understanding of the model, it would help if authors can assess and provide more information about the key parameters (other than the size of the network) that can be changed in the model to increase the number of retrievable item-context associations from 3 to, say, 4,5, or 6?

Reply: Thank you for pointing out this interesting question about capacity. Our model is capable of increasing the number of retrievable item-context associations. There are many key factors that contribute to forming more associations without observing the item-context decoupling effect. As already pointed out by the Reviewer, one key factor is the network size. Another model parameter that effectively boosts capacity is an increase in the associative binding connection probability (e.g., from 2% to 4%). Furthermore, increasing background unspecific noise can certainly improve capacity as items or contexts will be more excitable and can easier be retrieved. The semanization effect also affects capacity and can be controlled by plasticity parameters (please see our response to another Reviewer’s comment no. 6). Last but not least, strengthening associative binding by upregulating wgain can improve capacity (Table 1). Yet, there is an upper limit to wgain as extreme values can lead to implausible excitatory postsynaptic potentials (EPSPs). In response to the Reviewer’s comment we have added text into the Discussion section regarding improving the retrievable item-context associations in lines 542-550 (535-542 is also relevant). 24. Line 289-290: The statement about the failure of context recall could be rephrased as it seems repetitive.

Reply: In response to the Reviewer’s comment, we have rephrased the corresponding text "In fact, episodic loss in our network implies that no context is recalled despite the item memory activation.”, (lines 267-268). 25. Line 300-301: Item-context decoupling may only be one aspect of episodic memory as items memorized in a sequence within the same context can also form an episodic memory (for example Howard Eichenbaum lab’s odor-sequence task). So authors should be careful in replacing the observed item-context decoupling with terms such as semantization or loss of episodicity.

Reply: Thank you for pointing this out. By all means, real episodic memories have a temporal component, yet these episodes are much more complex and require multiple stimuli representations which collectively synthesize the episode. Consequently, realizing that semantization can have multiple manifestations (in simpler and more complex scenarios), which we do not explicitly address in our model, we mention this modeling limitation in the Discussion section (line 439-442) “Although our network model is limited to a simple item-context decoupling scenario, the proposed plasticity mechanistic explanation for the observed item-context decoupling effect may be generalized to support semantization in more complex scenarios in which other mechanisms may synergistically interact and contribute to decontextualization”. At the same time, we still claim that the item-context decoupling examined in our work is an important, though not the only, effect under the umbrella of semantization and loss of episodicity phenomena. 26. The definition of semantization is not clear in the field so far. Also, it is not known whether semantization is a biological limitation in retrieval or encoding. None of the previous studies, including the ones cited in the manuscript, have given conclusive evidence that loss of episodicity is associated with the weakening of associative synaptic weight distributions. So authors should clarify this limitation that the field of learning and memory is facing and the observed effects in this modeling study may not be the basis of behaviorally observed semantization. Authors do make a statement regarding this in discussion (line 432-433, I suppose) although it would be insightful for the reader to mention such limitation in this results section wherever conclusions are being drawn from data.

Reply: Thank you for this reflection, which we definitely share, as exemplified by the statement in the Discussion, kindly referred to by the Reviewer. Indeed, we cannot rule out other mechanisms that play important roles during memory retrieval and contribute to the semantization effect. We propose one explanation, which we find tangible and attractive from the perspective of general neuro-computational principles. Considering behaviourally observed manifestations of semantization it may be difficult to differentiate our hypothesis from other ones, which the Reviewer alludes to. In this light, it seems hard to make a meaningful statement in the Results section about the validity of our computational hypothesis in relation to the existing behavioral data beyond what we already demonstrate. The fact that there could be other explanations for the presented data and that the proposed line of reasoning has limitations, intrinsically imposed by the field of memory and learning, as the Reviewer mentions, is truly a matter suitable for a more general discussion rather than the Results section where the focus is on strictly modelling findings. Therefore we have decided to strengthen that debatable aspect in the Discussion section in the following way: lines (442-445) “Admittedly, our hypothesis does not exclude other seemingly coexisting phenomena and mechanisms supporting memory retrieval that may facilitate semantization over time, e.g. reconsolidation or systems consolidation due to sleep or aging (Friedrich et al., 2015)”. 27. Fig 3A: Why do item and context attractors (solid red lines) do not stop/oppose item-context decoupling?

Reply: The item-context decoupling relies on the associative connections that are subjected to plasticity. Consequently, it is the associative binding (Fig. 3A, dashed lines) that becomes weaker over the increasing number of associations and drives the semantization effect, not the pre-loaded within-network recurrent projections (solid red lines) supporting items and context memories in their respective networks. Recurrent within-network connectivity facilitates the activation of the full attractor memory once it is either cued (cued item or context paradigm) or indirectly stimulated by the associative binding through the other network. When the input current delivered from the associative binding is not sufficient, no attractor activation can be triggered and potentially oppose item-context decoupling. 28. Authors should discuss whether previous literature suggests decoupling arises due to inefficient retrieval of memories or inefficient encoding of memories?

Reply: We value an opportunity to strengthen the Discussion in line with the Reviewer’s comments. In fact we address it in the context of the Reviewer’s comment no. 26. 29. Fig 3B: During behavior, every item is always experienced in some context. So the model should incorporate partial context-cues along with items to closely resemble behavior. What would the model output be if each item is cued along with partial context cue, even for 4-association case?

Reply: Thank you for the insightful question. Typically, in Remember/Know (R/K) memory tasks, as in Opitz (2010), participants learn item-context pairs and later during recall are requested first to recognize items (“Know”) and secondly to recall any contextual information previously encoded with the corresponding item (“Remember”). Therefore, items can be presented without a meaningful context during test, i.e., without the type of context that is asked to be retrieved. So, the trial structure and the way we cue are behaviourally relevant to the modelled task (Opitz, 2010). The use of partial cue for the congruent context (which is requested to be retrieved) would increase its recall rate, as the partial cue itself would activate and complete the context attractor (“pattern completion”). Yet, this manipulation would contaminate the role of associative binding in recall. Also, this would not be a Remember/Know test. 30. Fig 3C and 3F: Which factors lead to relatively higher recall for 4-associations when context is cued (3F) as compared to when item is cued (3C)?

Reply: The Context network contains 10 different contexts while the Item network stores 4 items. This asymmetry creates different levels of local competition between memory patterns in each individual network. Consequently, we attribute the differences referred to by the Reviewer to these asymmetries between the two networks. Importantly, the BCPNN model is less vulnerable (sensitive to these asymmetries) than STDP because of the synaptic weight normalization effect, intrinsic to the definition of the BCPNN learning (see eq. 8). We do not explicitly raise this point in the manuscript since this effect has limited relevance to the actual semantization phenomenon. 31. Fig 4B-4D: Why is there a difference in cued recall for one association case between item-cue (4B) and context-cue (4D)? Why this difference is not observed in BCPNN model?

Reply: We agree with the Reviewer that this is a complex effect. To understand this imbalance, we should think of the learned weight matrix between 4 items and 10 contexts. Some items have several positive connections to items, some only one. This asymmetry generally leads to these observed imbalances, which are less prominent for the BCPNN model network (please see our response to the preceding comment no. 30). For deeper insights it helps to start with the four-association case. Cuing an item with four associated contexts initially recruits some extra spikes from all four previously coactivated contexts (see yellow spikes in the context network right after the onset of the yellow item cue in Figure 4A, ∼8800 ms). Even though only one context memory pattern eventually wins the local competition, the excitation from the other 3 weakly activated context patterns towards the associated item pattern boosts activation/retrieval (and even prolongs the activation of the cued item, also visible in thet spike raster in Figure 4A). In the case of a single association the activated (blue) item is not accompanied by any such extra spikes since all but one contexts have been learnt to be anti-correlated with the item (1 co-active / 9 competing contexts). However, when we cue the single association (blue) by a context rather than by an item pattern, this effect largely disappears as the Item network is much smaller (1 co-active / 3 locally competing items). The BCPNN is not affected so much by imbalances in the number of co-active and competing assemblies (representing patterns) in small networks, because the weight learning includes a normalization by estimates of pre- (Pi) and post-synaptic (Pj) firing rates: Pij/(Pi*Pj)) [see eq. 8]. The normalization renders BCPNN more robust than STDP in such cases. We expect these finite size-dependent phenomena to vanish in large-scale networks, where activations are much sparser overall such that relative differences disappear. Since the effects discussed here are not directly related to the concept of semantization and can be thus considered as secondary findings, we have decided not to expound these complex details (potentially obscuring the main thread) in the manuscript. 32. Fig 6: Is the final readout spike at 11sec a spontaneous spike? Why does it occur at 11sec?

Reply: Thank you for pointing this out. The readout spike at 11 sec is not spontaneous, but it is induced for updating the weight before final read-out. The neural simulator that we use (NEST) updates synaptic weights upon arrival of a pre-synaptic spike, that is synaptic weights are updated when they are about to be used for signal transmission. This spike serves only at updating the weights in the microcircuit model, and this readout spike has no effect on the statistical difference for the already converged weights presented in Fig. 6. 33. Line 399: What happens when kappa is increased to kappa-boost for one item-four context scenario?

Reply: Thank you for this relevant question about the kappa parameter. Upregulating plasticity (kappa boost) for an item-context pair of the 4-association case enhances recollection for the biased context when the item is cued, while the other non-biased contexts are suppressed (data not shown) in an analogous way as in Fig. 7B . So, we observe a similar behavior as in the 3-association case presented in Fig. 7. 34. Fig 7A: Why do the 4-association (yellow) and 2-association (orange) bars change from normal to biased conditions? Why 1- (blue) and 2-association (green) bars don’t change from normal to biased scenarios?

Reply: We should clarify that the 2-association case is not reflected with the orange bar, but with the green one. The single- and 2-association bars do not change from the normal to the biased scenario. The 3-association changes drastically, as expected, since one of its item-context pairs is encoded with boost plasticity (upregulated kappa). The 4-association case drops by ca. 5% and it is not statistically different between normal and biased scenarios. This minor score difference for the 4-association case is attributed to the background noise, which targets items and context throughout simulation. Only the 3-association case is expected to be significantly affected, as presented in Fig. 7A. 35. Fig 8: How does recall change from 4-context cued recall to 3- or 2-context cued recall?

Reply: A nearly decontextualized item (4-association scenario) could be retrieved with higher score by activating all of its 4 associated contexts sequentially. Similarly for the 2- or 3-context cued recall, the associated item retrieval score increases as more evidence is provided through the activation of all of the possible associations. Therefore, extra evidence boosts and completes retrieval. 36. Line 493: It’s not clear what authors mean by any item can be a context. Do they mean any item network can encode context or something else?

Reply: We appreciate this comment - we definitely agree that this sentence may sound somewhat confusing to the reader. In response to the Reviewer’s comment we have rephrased this sentence in the manuscript (lines 484-489) “We assume that familiarity recognition is simply characterized by lack of contextual information, yet the distinction we make between the Context and Item networks is arbitrary. Memory patterns stored in the Context network are referred to as contexts and those in the Item network as items. From the perspective of the network’s architecture, items and contexts have representations of the same nature - non-overlapping sparse distributed patterns.” 37. Line 506: It may make the sentence more clear if it is modified to, “Models of long-term consolidation suggest that retrieval of richly contextualized memories...”

Reply: Thank you for the suggestion, we appreciate the better reformulation of the sentence and we have thus updated the manuscript accordingly (line 571-572) “Models of long-term consolidation suggest that retrieval of richly contextualized memories become more generic over time”. References Barth AL, Poulet JF (2012) Experimental evidence for sparse firing in the neocortex. Trends in neurosciences 35:345-355.

Brette R, Gerstner W (2005) Adaptive exponential integrate-and-fire model as an effective description of neuronal activity. Journal of neurophysiology 94:3637-3642.

Chrysanthidis N, Fiebig F, Lansner A (2019) Introducing double bouquet cells into a modular cortical associative memory model. Journal of computational neuroscience 47:223-230.

DeFelipe, J., Ballesteros-Yanez, I., Inda, M.C., Munoz, A. (2006). Double-bouquet cells in the monkey and human cerebral cortex with special reference to areas 17 and 18. Progress in Brain Research, 154, 15-32.

Fiebig F, Herman P, Lansner A (2020) An indexing theory for working memory based on fast

Hebbian plasticity. eneuro 7.

Friedrich M, Wilhelm I, Born J, Friederici AD (2015) Generalization of word meanings during infant sleep. Nature communications 6:1-9. Henry Markram, Maria Toledo-Rodriguez, Yun Wang, Anirudh Gupta, Gilad Silberberg, and Caizhi Wu. Interneurons of the neocortical inhibitory system. Nature reviews neuroscience, 5(10):793-807, 2004.

Mountcastle VB (1997) The columnar organization of the neocortex. Brain: a journal of neurology 120:701-722. Opitz B (2010) Context-dependent repetition effects on recognition memory. Brain and Cognition 73:110-118.

Parisien, Christopher and Anderson, Charles H and Eliasmith, Chris (2008) Solving the problem of negative synaptic weights in cortical models. Neural computation 6: 1473-1494 Peters, A., & Yilmaz, E. (1993). Neuronal organization in area 17 of cat visual cortex. Cerebral Cortex, 3, 49-68.

Tully PJ, Hennig MH, Lansner A (2014) Synaptic and nonsynaptic plasticity approximating probabilistic inference. Frontiers in synaptic neuroscience 6:8. Thomson AM, West DC, Wang Y, Bannister AP (2002) Synaptic connections and small circuits involving excitatory and inhibitory neurons in layers 2-5 of adult rat and cat neocortex: triple intracellular recordings and biocytin labelling in vitro. Cerebral cortex 12:936-953.

Tsodyks MV, Markram H (1997) The neural code between neocortical pyramidal neurons depends on neurotransmitter release probability. Proceedings of the national academy of sciences 94:719-723.

Wang Y, Markram H, Goodman PH, Berger TK, Ma J, Goldman-Rakic PS (2006) Heterogeneity in the pyramidal network of the medial prefrontal cortex. Nature neuroscience 9:534-542.

Yoshimura Y, Callaway EM (2005) Fine-scale specificity of cortical networks depends on inhibitory cell type and connectivity. Nature neuroscience 8:1552-1559.
